# Development of high transferability cpSSR markers for individual identification and genetic investigation in Cupressaceae species

**DOI:** 10.1002/ece3.4053

**Published:** 2018-04-20

**Authors:** Li‐Sha Huang, Yan‐Qiang Sun, Yuqing Jin, Qiong Gao, Xian‐Ge Hu, Fu‐Ling Gao, Xiao‐Lei Yang, Ji‐Jun Zhu, Yousry A. El‐Kassaby, Jian‐Feng Mao

**Affiliations:** ^1^ Beijing Advanced Innovation Center for Tree Breeding By Molecular Design National Engineering Laboratory for Tree Breeding Key Laboratory of Genetics and Breeding in Forest Trees and Ornamental Plants Ministry of Education College of Biological Sciences and Technology Beijing Forestry University Beijing China; ^2^ Economic Forest and Seeding Management Station in Henan Province Zhengzhou China; ^3^ National Tree Breeding Station for Platycladus orientalis in Jiaxian Forest Farm of Jiaxian County Henan China; ^4^ Department of Forest and Conservation Sciences Faculty of Forestry The University of British Columbia Vancouver BC Canada

**Keywords:** chloroplast SSRs, Cupressaceae, genetic diversity, haplotype, mating system, paternity test

## Abstract

Given the low substitution rate in plastomes, the polymorphic and codominant nature of chloroplast SSRs (cpSSRs) makes them ideal markers, complementing their nuclear counterpart. In Cupressaceae, cpSSRs are mostly paternally inherited, thus, they are useful in mating systems and pollen flow studies. Using e‐PCR, 92 SSR loci were identified across six Cupressaceae plastomes, and primers were designed for 26 loci with potential interspecific transferability. The 26 developed cpSSRs were polymorphic in four genera, *Platycladus*,* Sabina*,* Juniperus,* and *Cupressus* and are suitable for Cupressaceae molecular genetic studies and utilization. We genotyped 192 *Platycladus orientalis* samples from a core breeding population using 10 of the developed cpSSRs and 10 nuclear SSRs, and these individuals were identified with high confidence. The developed cpSSRs can be used in (1) a marker‐assisted breeding scheme, specifically when paternity identification is required, (2) population genetics investigations, and (3) biogeography of Cupressaceae and unraveling the genetic relationships between related species.

## 1 INTRODUCTION

The cypress family, Cupressaceae, is the largest extant conifer family in terms of genera and the third largest in terms of species. The family includes 27 genera (17 of which are monotypic), with 142 species in total. Cupressaceae is also the most widely distributed gymnosperm family, present on all continents except Antarctica. Many of its species are vital sources of timber, and several genera are also important in ecological conservation and horticulture. *Platycladus*, which contains only one species, *Platycladus orientalis*, is native to China, Korea, and the Russian Far East (Cheng & Fu, 1978; Hu, Jin, Wang, Mao, & Li, 2015). As a pioneer species, *P. orientalis* is often used in ecological restoration projects (Jiang, Shi, Niu, & Yue, 2009; Li, He, & Ren, 2011). *P. orientalis* has a remarkable capability to absorb and accumulate atmospheric pollutants and heavy metal soil pollutants (Chu, 2012); thus, it is widely used for ecological remediation in densely populated cities in north China. Despite its importance, to date, little is known about this species’ population genetic diversity; however, scant nuclear genetic markers information is available (allozymes: (Xie, Dancik, & Yeh, 1992); AFLP: (Wang, Xing, Tang, & Feng, 2011); SSRs: (Jin et al., 2016)), all revealing low genetic diversity. Understanding *P. orientalis* population genetics and the extent of diversity in its germplasm collections are critical for the development of sound conservation and breeding strategies. The implementation of marker‐assisted breeding methods such as “Breeding without Breeding,” which is based on paternity assignment and pedigree reconstruction, requires the availability of diagnostic genetic markers (El‐Kassaby & Lstibůrek, 2009; El‐Kassaby, Cappa, Liewlaksaneeyanawin, Klápště, & Lstibůrek, 2011).

The uniqueness of organelles genomes has been useful in plants population genetics and evolution due to their nonrecombinant nature and uniparental inheritance (Birky, 1995). In plants, the mitochondrial genome is highly variable and has high levels of intramolecular recombination (Olmstead & Palmer, 1994), rendering them challenging research tool (Provan, Powell, & Hollingsworth, 2001). However, the plastome generally lacks recombination and often has the potential to develop genetic makers (Ebert & Peakall, 2009). Furthermore, plastids are paternally inherited in most Cupressaceae species (Sakaguchi, Tsumura, Crisp, Bowman, & Isagi, 2014), making them ideal markers for monitoring gene flow via pollen and effective breeding and phylogeny tools (Vendramin, Lelli, Rossi, & Morgante, 1996).

The high level of substitution conservation in the plastome confirms the feasibility of using cpSSRs to reveal their genome diversity. CpSSRs, as genetic markers, were first developed by Powell (Powell, Morgante, Andre et al., 1995), highlighting their high polymorphism and codominant inheritance, making them attractive genetic markers, coupled with the fact that only few loci are needed to identify unique genotypes (Schlotterer & Tautz, 1992). Plastome SSR loci are often distributed throughout the noncoding regions and show greater sequence variation than the coding regions and are characterized by low evolutionary rate and an almost nonexistent recombination rate (Powell, Morgante, Andre et al., 1995; Powell, Morgante, McDevitt, Vendramin, & Rafalski, 1995; Powell, Machray, & Provan, 1996), making them ideal molecular tools to complementing their nuclear markers counterparts (Huang et al., 2015; Provan et al., 2001). CpSSRs have been found to be transferable among related species because the regions flanking the cpSSR loci are conserved (Pan et al., 2014), thus, have the potential to evaluate both inter‐ and intraspecific variability (Powell, Morgante, Andre et al., 1995; Powell, Morgante, McDevitt et al., 1995).

Here, we identified 92 cpSSRs loci in the plastomes of Cupressaceae species and developed 26 transferable SSR markers. The primers were screened for intraspecific polymorphism across different genera, and discrimination rate of the polymorphic loci was evaluated by genetic investigation of a core breeding population of *P. orientalis*. This study presents a set of polymorphic cpSSR markers which are transferable across diverse genera of Cupressaceae and demonstrates their value for genetic discrimination and diversity studies in this family.

## 2 MATERIALS AND METHODS

### 2.1 Study species, sample collection, and DNA extraction

To evaluate the polymorphism and interspecific transferability of the cpSSR markers with the designed primers, we sampled 48 individual trees representing *Platycladus orientalis* (*n* = 24), *Sabina chinensis* (*n* = 8), *Juniperus formosana* (*n* = 8), and *Cupressus torulosa* (*n* = 8). These trees were collected from different regions of China and planted in the National Tree Breeding Station for *Platycladus orientalis* in Jiaxian (Forest Farm of Jiaxian County, Henan, China) and the Institute of Forestry and Pomology (Beijing Academy of Agriculture and Forestry Sciences).

Additionally, we characterized the genetic variation in the plastomes of a core breeding population (*n* = 192 parent trees) of *P. orientalis* growing in a seed orchard located in the National Tree Breeding Station for *Platycladus orientalis* in Jiaxian. The seed orchard was established in the mid‐1980s’, representing a wide range of *P. orientalis*. The sampled 192 parent trees originated from three populations (northern, southwestern, and eastern) covering the northern to southern Henan province (lat.: 33.33° (N) to 35.42° (N), long.: 112.17° (E) to 113.51° (E)); the populations are geographically divided by the Yellow River and the Taihang Mountains (the sampled trees 21–136 from a specific forest stand are regarded as a population) (Table 1).

**Table 1:** Origins of the 192 sampled *Platycladus orientalis* individuals

Population	Sample size	Longitude (E)	Latitude (N)
North	138	113.51°	35.42°
East	21	113.43°	33.33°
Southwest	33	112.17°	34.36°

### 2.2 SSR detection and primer design

The GMATA2.1 (Wang & Wang, 2016) program for SSR mining and marker development was employed to identify SSR loci in the plastomes of six species *Cupressus gigantean* (GenBank ID: KT315754.1) (Li, Guo, & Zheng, 2016), *Cupressus sempervirens* (GenBank ID: KP099643.1), *Juniperus monosperma* (GenBank ID: NC_024022.1), *Juniperus bermudiana* (GenBank ID: KF866297.1), *Juniperus scopulorum* (GenBank ID: NC_024023.1), and *Juniperus virgiana* (GenBank ID: NC_024024.1) (Guo et al., 2014) using constraints of more than five repeats and a motif length between 2 and 10 bp. Electronic PCR (e‐PCR) refers to the process of recovering unique sequence‐tagged sites in DNA sequences by searching for subsequence that closely match the PCR primers and have the correct order, orientation, and spacing that they could plausibly prime the amplification of a PCR product of the correct molecular weight (Schuler, 1997). Therefore, e‐PCR presents a good tool to evaluate the designed primer pairs for interspecific amplification. We performed e‐PCR to select potentially interspecific transferable SSRs. The selected primers, which showed interspecific transferability in e‐PCR and potential polymorphism, were screened with M13 attached to the 5′ end of the forward primer. Primer3 (Rozen & Skaletsky, 1999) was employed to design primers for the loci with the following constraints: amplicon size of 120 ~ 400 bp, annealing temperature of about 60°C, and flanking region size ≤2,000 bp. Comparison of DNA sequences from the six species up to one hundred kb long and visualization of the alignments with annotations was generated using VISTA (Frazer, Pachter, Poliakov, Rubin, & Dubchak, 2012; Mayor et al., 2000). The feasibility of primer amplification of these 26 e‐PCR‐selected primer pairs was examined in other chloroplast genomes of seven Cupressaceae species from six genera by e‐PCR.

### 2.3 Primer screening and detection of cpSSR polymorphisms

In the initial screening, we amplified the designed markers from mixed DNA of each species across different genera. In a further screening, DNA from individual trees was used to amplify the cpSSR markers which showed clear amplification in the initial step, and polymorphism levels were determined by fluorescent‐based capillary electrophoresis with an ABI 3730 sequencer. PCRs were carried out in a total volume of 20 μl including 10 μl 2 × Taq PCR Mix, 4 μl (4 pmol) fluorescent‐dye‐labeled M13 primer, 2 μl (10 ng) genomic DNA, and 4 μl (4 pmol) mixed complementary forward and reverse primer. PCR amplifications were performed using Bio‐Rad T100^™^ Thermal Cycler and Bio‐Rad S1000^™^ Thermal Cycler with the following profile: 4‐min denaturing at 94°C; 20 cycles of 30‐s denaturing at 94°C, 30‐s annealing at 60°C (−0.5°C per cycle), and 45‐s extension at 72°C; 20 cycles of 30‐s denaturing at 94°C, 30‐s annealing at 50°C, and 45‐s extension at 72°C, with a final extension step of 72°C for 5 min. The amplification products were separated on a 1.0% agarose gel at 150 V for 15 min and visualized by GoldView staining.

The number of observed alleles (*N*_a_), the effective number of alleles (*N*_e_), Shannon index (*I*), diversity index (*h*), and the unbiased diversity (*uh*) were calculated using GenALEx version 6.5 (Peakall & Smouse, 2012).

### 2.4 CpSSR evaluation with a core breeding population

We selected 24 primers for genotyping the 192 plastomes of *P. orientali*s, after excluding two primer pairs (N19 and N29) which displayed low polymorphism in the initial screening. Nuclear SSR polymorphism data for the same sample set were collected from the previous study (Jin et al., 2016). GeneCap version 1.4 (Wilberg & Dreher, 2004) was used to distinguish the different haplotypes by contrasting all alleles. Principal component analysis (PCA) was performed using the “adegenet” package (Jombart, 2008) to reference the redistribution of populations in R (Ihaka & Gentleman, 1996). The minimum genetic distance matrix was calculated using populations‐1.2.32 (http://bioinformatics.org/~tryphon/populations/), and the matrix was used to generate a molecular phylogenetic tree in Splitstree 4.14.2 (Huson, 1998). The neighbor network graph was generated by Splitstree 4.14.2, and the phylogenetic tree graph was displayed by FigTree v1.4.2 (Rambaut, 2012).

## 3 RESULTS

### 3.1 SSR analysis and primer design

Ninety‐two SSRs with more than five repeats and a motif length ranging between 2 and 10 bp were identified in the plastomes of six species of two genera, *Cupressus* and *Juniperus*. Dinucleotide repeats were the most abundant, with a count of 73 (79.35%), followed by 13 tri‐, 4 nona‐, 1 tetra‐, and 1 hepta‐nucleotide repeats (14.13%, 4.35%, 1.09%, and 1.09%, respectively) (Table S1). The locations of these 92 loci are presented in Figure [Link], [Link], [Link], [Link], [Link], [Link]. In the six species plastomes, the most common dinucleotide repeat was (AT/TA)_n_ and the most common trinucleotides were (AGA/TCT)_n_ and (TTC/GAA)_n_.

**Figure 1:**
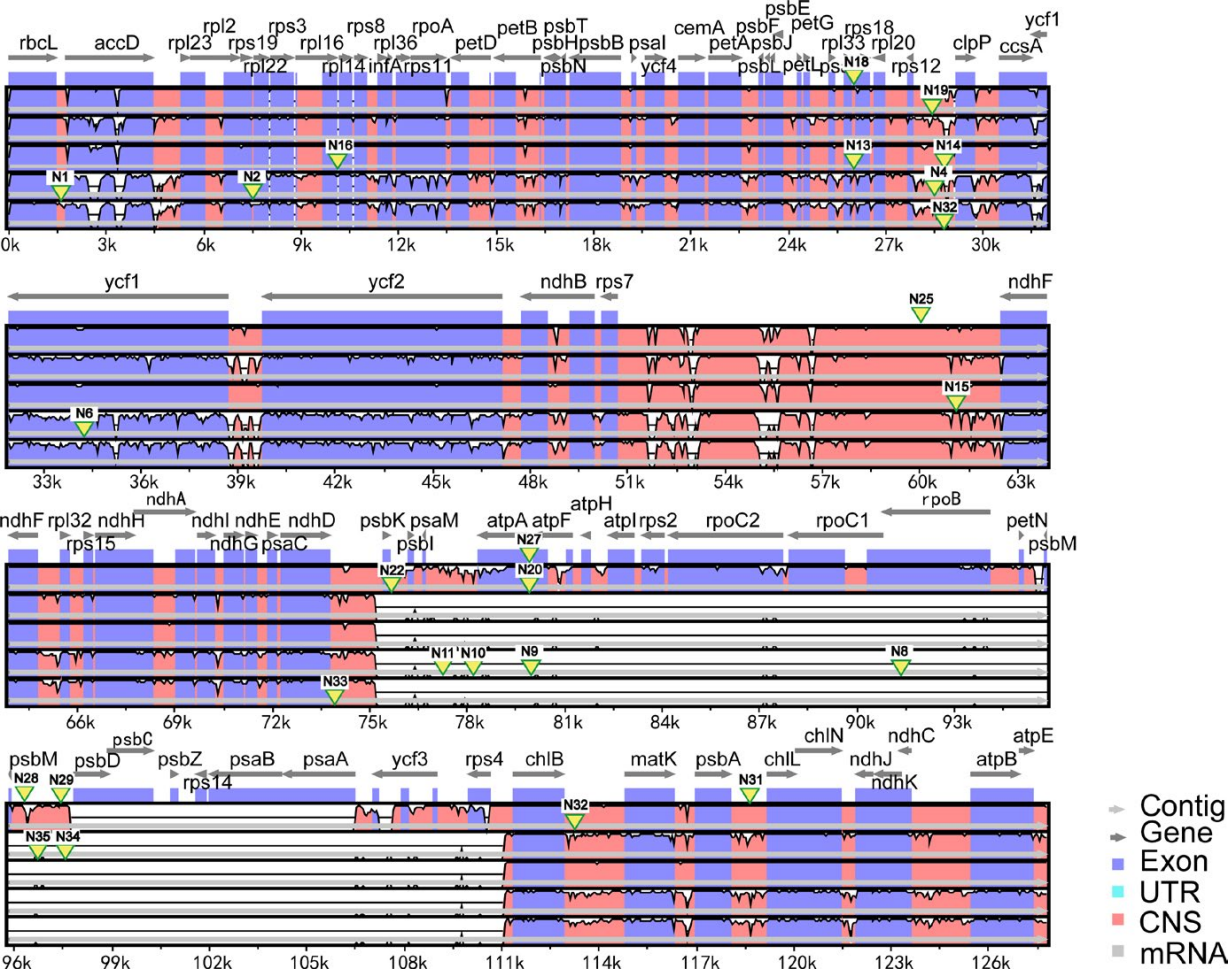
Comparison of plastomes from six species and the locations of the designed cpSSRs. The SSR loci of 26 primers were marked in the figure. This figure uses the chloroplast genome of *Juniperus scopulorum* (GenBank ID: NC_024023.1) as the reference, so it acts as the *X* axis. Alignment 1–5 (rows in each block of alignment) denotes *Juniperus bermudiana*,* Juniperus monosperma*,* Juniperus virginiana*,* Cupressus sempervirens,* and *Cupressus gigantean*, respectively. CNS, conserved noncoding sequences

After SSR detection, we performed e‐PCR. With e‐PCR, we selected 26 potentially interspecific transferable primer pairs which targeted 22 di‐, 3 tri‐, and 1 nona‐nucleotide SSRs. Among these 26 SSRs, 23 occurred in intergenic spacer regions, two were in coding sequence regions (N6 in ycf1 and N8 in rpoB), and one (N10) in an intron of tmG‐UCC (Table 2, Figure 1).

**Table 2:** Primer pairs of 26 cpSSRs designed by mining the plastomes of six species

Species	Sequences	ID	Location	*T*_m_	Product size	Repeat motif
*Cupressus gigantean*	F: TTCTAGCTCGCACCCAAACT R: TTGTTTCGCCGATATGTTCA	N1	rbcL/accD IR	60.04	209	(AC)_6_
*Cupressus gigantean*	F: TGGTCATACCATTGCTGTTCA R: TGGGCTACTCTACGTGCTTT	N2	rps19/rp122 IR	58.82	388	(AT)_5_
*Cupressus gigantean*	F: TCGGAAGAAGAAGATGATATGTAGC R: CCCCAGATATGGAACTTTTGG	N4	rps12 Exon1/clpP IR	60.33	138	(AAGGACAAA)_5_
*Cupressus gigantean*	F: GGGAACAACCAGAATTGGAA R: GCCACTTTTATGGCACGACT	N6	ycf1	59.95	365	(TA)_5_
*Cupressus gigantean*	F: GGGAAGCGGAAAGCTATTTT R: GGTAATCCACAGCAGCCAAT	N8	rpoB	59.83	265	(AT)_6_
*Cupressus gigantean*	F: CAAATTTCTCGCCAAGCTGT R: TGATTTCATCGGGTCGAATA	N9	atpF Exon2/atpA IR	59.65	210	(TA)_12_
*Cupressus gigantean*	F: TCGGGAACGAAAGAGAAAGA R:ACATAGATGTTATGGAGCAGAGC	N10	trnG‐UCC intron	58.74	290	(CA)_5_
*Cupressus gigantean*	F: TCCAATCTAGAACATCTCATCCAG R: CAGTGCTCTACCTAATCTGAAAGC	N11	trnG‐UCC/psaM IR	59.21	226	(AT)_6_
*Cupressus sempervirens*	F: TCGCCGCAATACTCCTAATC R: ATTCCGAAAAGATGGCTTCA	N13	rpl33/rps18 IR	59.92	277	(AT)_5_
*Cupressus sempervirens*	F: TCCATATCTGGTGGACAGGA R: GGGTTTTGGTCTTCTTCTTCG	N14	rps12 Exon1/clpP IR	59.49	141	(AT)_6_
*Cupressus sempervirens*	F: CCAGGTCGAGACAAGTGGAT R: GAAACCAATGCCCTAAGCAA	N15	rrn16/trnI‐GAU IR	60.09	350	(AT)_6_
*Cupressus sempervirens*	F: ATTCGATCCCTATCCGGTCT R: ACCAGAGCCATCAACCACTC	N16	psbZ/trnS‐UGA IR	59.93	253	(AT)_5_
*Juniperus bermudiana*	F: AAAATCGCCGCAATACTCCT R: ATTCCGAAAAGATGGCTTCA	N18	rpl33/rps18 IR	60.29	276	(AC)_6_
*Juniperus monosperma*	F: CGGACCTACCGACAGAACTC R: CCGAAGAAATAAGAAGCTGTATAGG	N19	rps12 Exon1/clpP IR	59.35	369	(AGA)_6_
*Juniperus monosperma*	F: CTTGCTCCTAGCCATGAAAA R: TGATTTCATCGGGTCGAATAG	N20	atpF/atpA IR	59.01	277	(AT)_5_
*Juniperus monosperma*	F: CTGTGATGCCGTTGATATTGA R: TGCCCATTATCCCTCTGTTC	N22	psbI/psbK IR	59.73	334	(CA)_5_
*Juniperus monosperma*	F: TCCTCTGCGATCTTTTATAGGG R: GGGAAGGATTGTTGGATTGA	N23	chlB/trnK‐UUU IR	59.72	270	(AT)_6_
*Juniperus bermudiana*	F: CCTCATACGGCTTCTCGTTC R: AAAATGAACCCCGAAGGATT	N25	rps12/trnV‐GAC IR	59.74	271	(TA)_5_
*Juniperus bermudiana*	F: TGATTTCATCGGGTCGAATA R: CAAATTTCTCGCCAAGCTGT	N27	aptA/aptF IR	59.65	314	(AT)_7_
*Juniperus bermudiana*	F: GAGATGAACGCAGAGCGTAA R: TGTTTGAGCGATTCCTACCC	N28	psbM/trnD‐GUC IR	59.63	203	(AT)_7_
*Juniperus bermudiana*	F: GCCATGGTAAGGCGTAAGTC R: TCAGTCAATGGGTTAGGTTCA	N29	trnT‐GGU/psbD IR	58.83	365	(TA)_5_
*Juniperus bermudiana*	F: GTAGCCAAGTGGTTCCAAGG R: CAATCTTACCGTCGATTCAGC	N31	trnH‐GUG/chlL IR	59.66	254	(AT)_5_
*Juniperus scopulorum*	F: GGACGGGAAAGGAAAAGAA R: TGGTATTCTTCTGGGTTTTGG	N32	rps12 Exon1/clpP IR	59.01	219	(AT)_5_
*Juniperus scopulorum*	F: CTGTTCCCCTGTGCATCATA R: AGGAGGAAAATCCGTTGGTT	N33	trnF‐GAA/trnL‐UAA IR	59.66	350	(TCT)_5_
*Juniperus virgiana*	F: TCAGTCAATGGGTTAGGTTCA R: GCCATGGTAAGGCGTAAGTC	N34	PsbD/trnT‐GGU IR	58.83	365	(TCT)_5_
*Juniperus virgiana*	F: TGTTTGAGCGATTCCTACCC R: GAGATGAACGCAGAGCGTAA	N35	trnD‐GUC/psbM IR	59.63	203	(AT)_6_

*T*_m_, melting temperature; location refers to the genomic location in the plastomes of the six species (*Cupressus gigantean*,* Cupressus sempervirens*,* Juniperus monosperma*,* Juniperus bermudiana*,* Juniperus scopulorum*, and *Juniperus virgiana*); IR, intergenic region.

### 3.2 Chloroplast SSR amplification and polymorphism detection

Initial screening via electrophoresis showed that amplicons were obtained for 24 of the 26 e‐PCR‐selected primer pairs, and amplification of two primer pairs (N14 and N23) was detected only in *P. orientalis*,* S. chinensis*, and *J. formosana*, and not in *C. torulosa*. Further analyses of the cpSSRs were carried out in these four species.

In the second screening step, the amplicons of most of the primer pairs were monomorphic in the *C*. *torulosa*, while most of pairs showed polymorphism in *J*. *formosana* samples. These results may be due to the different degree of genetic variation among the samples. Seven primer pairs (N2, N11, N13, N16, N18, N25, and N35) demonstrated polymorphism across all four species. Primer pairs N19, N27, and N35 had the maximum effective number of alleles (*N*_e_ = 5.33), while primer pair N35 generally showed the highest levels of polymorphism across all samples (Table 3). The two loci (N6 and N8) from coding regions and one locus (N10) from an intron region displayed levels of polymorphism comparable with other loci.

**Table 3:** Characteristics of 26 cpSSR markers in 48 individuals of *Platycladus orientalis* (*n* = 24), *Sabina chinensis* (*n* = 8), *Juniperus formosana* (*n* = 8), and *Cupressus torulosa* (*n* = 8)

Marker ID	*Platycladus orientalis*					*Sabina chinensis*					*Juniperus formosana*					*Cupressus torulosa*				
	*N*_*a*_	*N*_e_	*I*	*h*	*uh*	*N*_*a*_	*N*_e_	*I*	*h*	*uh*	*N*_*a*_	*N*_e_	*I*	*h*	*uh*	*N*_*a*_	*N*_e_	*I*	*h*	*uh*
N1*	7	3.88	1.63	0.74	0.79	1	1.00	0.00	0.00	0.00	4	3.56	1.32	0.72	0.82	1	1.00	0.00	0.00	0.00
N2*	5	3.06	1.30	0.67	0.71	2	1.28	0.38	0.22	0.25	3	2.46	0.97	0.59	0.68	4	2.29	1.07	0.56	0.64
N4^#^	2	2.00	0.69	0.50	0.53	1	1.00	0.00	0.00	0.00	3	2.46	0.97	0.59	0.68	1	1.00	0.00	0.00	0.00
N6^~^	5	3.61	1.40	0.72	0.76	2	1.28	0.38	0.22	0.25	4	3.56	1.32	0.72	0.82	1	1.00	0.00	0.00	0.00
N8*	4	2.17	0.98	0.54	0.56	1	1.00	0.00	0.00	0.00	3	1.68	0.74	0.41	0.46	1	1.00	0.00	0.00	0.00
N9*	4	2.72	1.14	0.63	0.68	2	1.28	0.38	0.22	0.25	1	1.00	0.00	0.00	0.00	1	1.00	0.00	0.00	0.00
N10^#^	2	1.92	0.67	0.48	0.51	2	1.28	0.38	0.22	0.25	5	4.57	1.56	0.78	0.89	1	1.00	0.00	0.00	0.00
N11*	2	1.60	0.56	0.38	0.40	2	1.28	0.38	0.22	0.25	4	3.56	1.32	0.72	0.82	3	2.13	0.90	0.53	0.61
N13*	4	3.43	1.29	0.71	0.74	3	2.13	0.90	0.53	0.61	3	2.13	0.90	0.53	0.61	2	1.60	0.56	0.38	0.43
N14^~^	6	4.48	1.64	0.78	0.85	4	3.56	1.32	0.72	0.82	5	4.00	1.49	0.75	0.86	0	0.00	0.00	0.00	0.00
N15^^^	6	5.00	1.70	0.80	0.89	2	1.28	0.38	0.22	0.25	5	4.00	1.49	0.75	0.86	1	1.00	0.00	0.00	0.00
N16^#^	3	2.46	0.97	0.59	0.63	2	1.28	0.38	0.22	0.25	5	4.00	1.49	0.75	0.86	2	1.60	0.56	0.38	0.43
N18^#^	3	2.46	0.97	0.59	0.63	2	1.28	0.38	0.22	0.25	5	3.20	1.39	0.69	0.79	2	1.60	0.56	0.38	0.43
N19	1	1.00	0.00	0.00	0.00	2	1.28	0.38	0.22	0.25	6	5.33	1.73	0.81	0.93	1	1.00	0.00	0.00	0.00
N20*	3	2.46	0.97	0.59	0.63	2	1.28	0.38	0.22	0.25	5	4.57	1.56	0.78	0.89	1	1.00	0.00	0.00	0.00
N22^#^	2	1.80	0.64	0.44	0.46	2	1.28	0.38	0.22	0.25	4	2.91	1.21	0.66	0.75	1	1.00	0.00	0.00	0.00
N23^~^	3	2.31	0.94	0.57	0.64	1	1.00	0.00	0.00	0.00	2	1.28	0.38	0.22	0.25	1	1.00	0.00	0.00	0.00
N25^#^	4	2.72	1.14	0.63	0.68	2	1.28	0.38	0.22	0.25	3	2.13	0.90	0.53	0.61	2	1.28	0.38	0.22	0.25
N27*	2	1.28	0.38	0.22	0.23	2	1.28	0.38	0.22	0.25	6	5.33	1.73	0.81	0.93	1	1.00	0.00	0.00	0.00
N28^~^	4	1.81	0.89	0.45	0.49	3	1.68	0.74	0.41	0.46	5	4.57	1.56	0.78	0.89	0	0.00	0.00	0.00	0.00
N29	2	1.13	0.23	0.12	0.13	2	1.28	0.38	0.22	0.25	3	2.46	0.97	0.59	0.68	1	1.00	0.00	0.00	0.00
N31^^^	2	2.00	0.69	0.50	0.53	2	1.60	0.56	0.38	0.43	5	4.00	1.49	0.75	0.86	1	1.00	0.00	0.00	0.00
N32^~^	5	4.26	1.51	0.77	0.82	5	3.20	1.39	0.69	0.79	6	4.57	1.67	0.78	0.89	0	0.00	0.00	0.00	0.00
N33*	5	4.24	1.52	0.76	0.83	2	1.28	0.38	0.22	0.25	4	2.91	1.21	0.66	0.75	1	1.00	0.00	0.00	0.00
N34*	2	2.00	0.69	0.50	0.53	2	1.28	0.38	0.22	0.25	3	2.91	1.08	0.66	0.75	1	1.00	0.00	0.00	0.00
N35^^^	3	2.67	1.04	0.63	0.83	2	1.28	0.38	0.22	0.25	6	5.33	1.73	0.81	0.93	2	1.38	0.45	0.28	0.33

*N*_a_, observed number of alleles; *N*_e_, effective number of alleles; *I*, Shannon's information index; *h,* diversity; *uh,* unbiased diversity; *, #, ~, and ^ indicating polymorphic, monomorphic, low amplification rate, low specificity markers, respectively, characterized in a core breeding population of 192 individuals of *P. orientalis*.

In addition, we also examined the feasibility of primer amplification of the 26 e‐PCR‐selected primer pairs in other chloroplast genomes of seven Cupressaceae species from six genera by e‐PCR. Eighteen primer pairs (N1, N2, N4, N6, N8, N9, N10, N11, N13, N16, N18, N19, N22, N25, N31, N33, N34, and N35) amplified successfully in the genome of *Cupressus jiangeensis*. Four pairs (N8, N9, N16, and N20) and three pairs (N2, N18, and N29) amplified successfully in the genomes of *Calocedrus formosana* and *Thujopsis dolabrata*, respectively. Five primer pairs (N1, N2, N9, N18, and N34), two pairs (N9 and N34), one pair (N18), and two pairs (N27 and N29) amplified successfully in the genomes of *Thuja standishii*,* Cunninghamia lanceolata*,* Calocedrus macrolepis,* and *Hesperocyparis glabra*, respectively (Table S2).

### 3.3 Application of cpSSR markers in genetic variation analysis

Among the 24 primer pairs characterized in the *P. orientalis* core breeding population (*n* = 192), ten pairs (N1, N2, N8, N9, N11, N13, N20, N27, N33, and N34) amplified well and showed polymorphism while the remaining were monomorphic and exhibited low amplification and specificity (Table 3). The performance of these ten primer pairs was uneven, and they displayed different degrees of polymorphism (Table 4). The core breeding population's genetic parameters were as follows: number of observed alleles (*N*_a_) ranged from 2 to 9, effective number of alleles (*N*_e_) ranged from 1.021 to 2.743, Shannon's information index (*I*) ranged from 0.058 to 1.294, diversity index (*h*) ranged from 0.021 to 0.635, and unbiased diversity (*uh*) ranged from 0.021 to 0.639 (Table 4). Interestingly, locus N8, from the rpoB gene coding region, displayed fairly high degree of diversity. Although only some of the loci produced lower polymorphisms, they nevertheless collectively presented a wide range of polymorphism. Ten polymorphic cpSSR loci revealed 134 unique genotypes in the 192 sampled trees. Statistical confidence for individual identification using these ten loci was moderate (*p*_ID_ = .0086). Thus, not every tree had a unique multilocus genotype, and 28 of the 134 genotypes were assigned to 2–7 trees, with a total of 86 trees sharing genotypes.

**Table 4:** Characterization of 10 polymorphic cpSSR loci from 192 individuals of *Platycladus orientalis*

Loci	Allele size (bp)	*N*_a_	*N*_e_	*I*	*h*	*uh*
N1	247, 255, 283, 313, 343, 371	6	2.743	1.181	0.635	0.639
N2	354, 356, 384, 416, 448	5	1.114	0.277	0.102	0.103
N8	279, 281, 283, 285, 287, 289, 291, 293, 299	9	2.447	1.294	0.591	0.595
N9	306, 308, 310, 312, 314, 316	6	1.229	0.455	0.187	0.188
N11	250, 252, 284, 292	4	1.417	0.533	0.294	0.296
N13	293, 299	2	1.051	0.116	0.048	0.049
N20	211, 213, 215, 217, 219, 221, 223, 225	8	1.395	0.678	0.283	0.285
N27	308, 310, 312, 314, 316, 218, 330	7	1.258	0.490	0.205	0.206
N33	359, 413, 419, 440	4	1.842	0.809	0.457	0.460
N34	362, 386	2	1.021	0.058	0.021	0.021

*N*_a_, observed number of alleles; *N*_e_, effective number of alleles; *I*, Shannon's information index; *h*, diversity; *uh*, unbiased diversity.

According to the polymorphism information of the ten loci, PCA partitioned 72.74 4.33, and 2.94% of the variance in the data along the first three axes, respectively, collectively accounting for 80.01% of the total variation. PCA did not show clear separation of the genotypes into discrete clusters (Figure S7). In the generated dendrogram, the 192 samples were grouped into seven main clades (Figure S8: note individuals color correspond to population origin).

**Figure 2:**
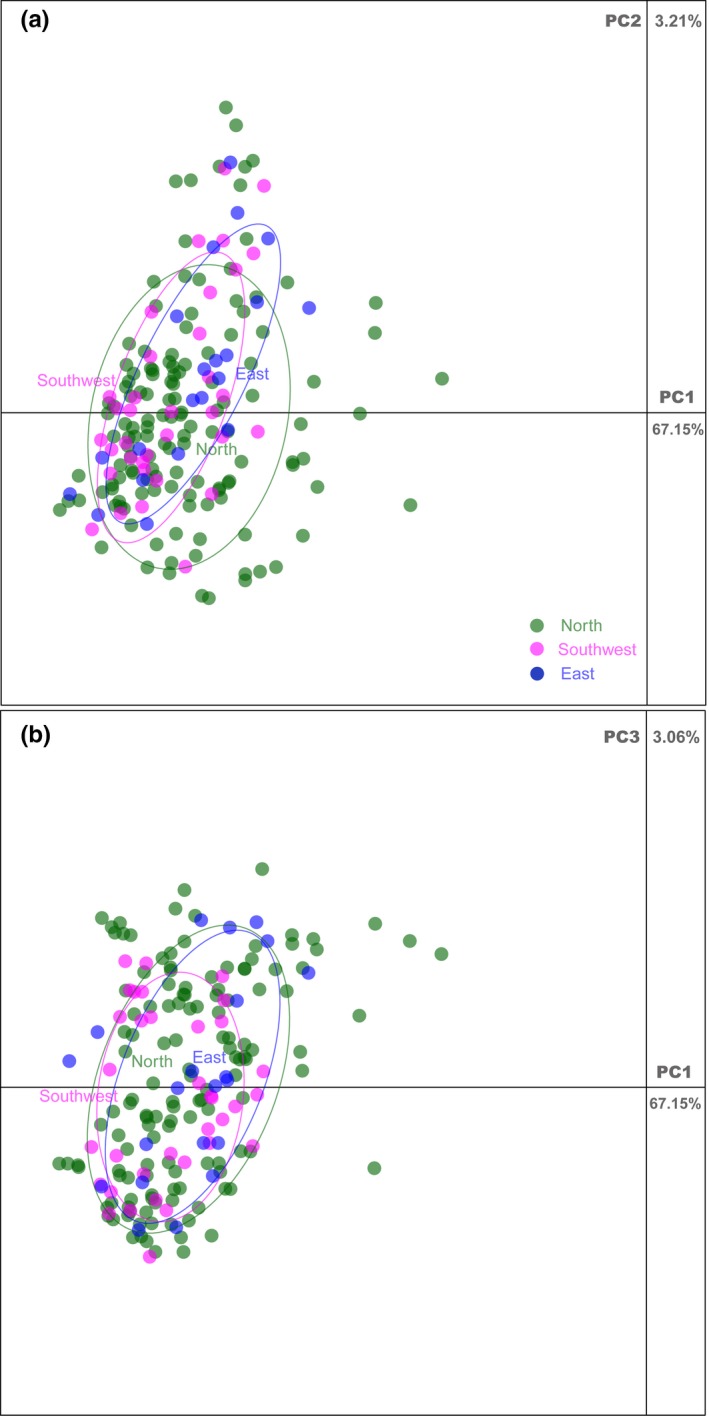
Principal component analysis (PCA) of *Platycladus orientalis* populations’ plastomes based on the polymorphism information of 10 cpSSRs and 10 nuclear SSRs

**Figure 3:**
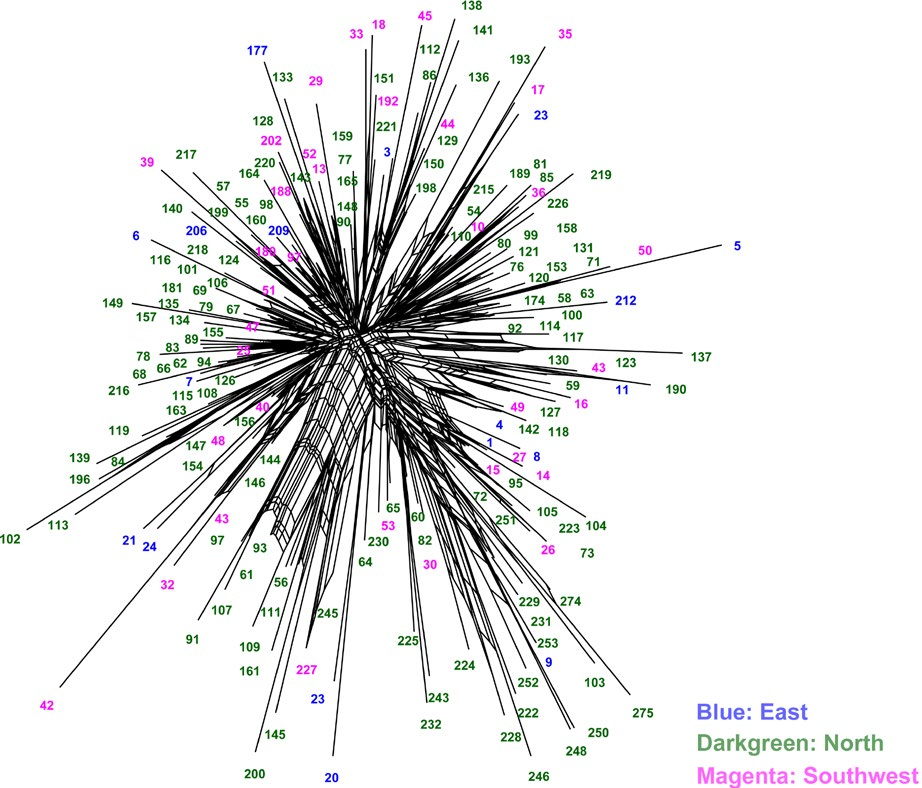
Neighbor network of *Platycladus orientalis* individuals. The trees were marked with different colors indicating the geographic information

We combined the ten polymorphic cpSSR loci with ten previously developed nuclear SSR loci (Jin et al., 2016) to further investigate the efficiency of these combined markers in individual discrimination and population genetic analysis. The statistical confidence for individual identification was high (*p*_ID_ = 0.0000545), and we obtained 192 unique genotypes corresponding to the 192 genotyped trees (i.e., each individual has a unique fingerprint). However, no clear genetic separation among the populations was observed in either the PCA, network, or phylogenetic tree. PCA partitioned 67.15, 3.21, and 3.06% of the variance along the first three components, respectively, and collectively accounting for 73.42% of the total variation (Figure 2). The neighbor network based on genetic distances among individuals produced a mixed network with no clear geographic structuring (Figure 3). All the sampled individuals were extensively mixed with each other and shared large amounts of recent admixtures (Figure 3).

## 4 DISCUSSION

To establish a sound cpSSR genotyping platform for Cupressaceae species, we developed 26 polymorphic and interspecific transferable cpSSR markers for Cupressaceae species by whole plastome analyses and experimental examination. We further assessed intraspecific chloroplast diversity of 192 *P. orientalis* samples and demonstrated high confidence for individual identification using cpSSR accompanying with small set of nuclear SSRs. The cpSSRs found in this study are valuable genetic markers for its high degree of interspecific transferability and polymorphism and encourage the use of cpSSRs in population genetic research and coniferous tree breeding. CpSSRs are ideal molecular tools complementing nuclear genetic markers in phylogenetic and population genetic studies (El‐Kassaby & Lstibůrek, 2009; El‐Kassaby et al., 2011) and can potentially be used to study mating systems and track gene flow for widespread and ecologically/economically important conifer species for which plastomes are paternally inherited (Vendramin et al., 1996).

### 4.1 E‐PCR is a critical step to predicate the transferable SSRs

The high quality, versatility, and applicability of our primers demonstrate the viability of the implemented method for developing SSR loci. We first identified and characterized SSRs along with gene features in each given genome/sequence (Wang & Wang, 2016). In the following step, simulated marker mapping (e‐mapping) was performed across all genomes/sequences using a forward e‐PCR algorithm, allowing evaluating the transferability of the cpSSRs and calculating the potential intergenomic/intersequence polymorphism of each developed SSR locus (Schuler, 1997). Finally, primers were designed for SSR loci were chosen for their transferability and potential polymorphism. The e‐PCR step is unique compared with other SSR markers development studies. The high rate of polymorphism attained in the present study (100%) indicates that e‐PCR may be a key step providing valuable information to increasing the rate of generating polymorphic SSR markers. The amplification of 26 pairs of primers detected by e‐PCR in seven Cupressaceae species (*Cupressus jiangeensis*,* Calocedrus formosana*,* Thujopsis dolabrata*,* Thuja standishii*,* Cunninghamia lanceolata*,* Calocedrus macrolepis,* and *Hesperocyparis glabra*) showed strong versatility of the primers and also provided important instructions for the use of molecular markers for these species.

### 4.2 Variation across the chloroplast genome

Formed through a process of mutation known as slippage replication, SSRs are believed to be a key source of genetic variation for plastomes (Provan et al., 2001). Codon sequences are highly conserved in chloroplasts, whereas intron and intergenic regions are variable. In the present study, we found that most of the polymorphic cpSSRs were located in intergenic or intron regions (23 loci in intergenic spacer regions and one in an intron), which is consistent with previous studies (Powell, Morgante, Andre et al., 1995; Powell, Morgante, McDevitt et al., 1995; Powell et al., 1996; Provan et al., 2001). The distribution of polymorphisms indicates that the genetic relationship between samples is the significant factor determining the variation in the number of repeats. Successful amplification demonstrated the viability of the primers, which were designed using the plastome of related species. Some primers may generate PCR products among unrelated species; however, because the loci are highly conserved, it is expected that there will be no polymorphism among unrelated species (Cheng et al., 2005). We also found that some of the primers failed to show polymorphism between species, which may result from the proximity of the cpSSR locus to a very conservative coding sequence.

Most interestingly, we found that the two loci from coding regions (N6 and N8) displayed polymorphism at a level comparable with other loci. N6 is located in the coding region of ycf1, one of the two largest ORFs in the plastome, which encodes the TIC214 protein involved in protein precursor import into chloroplasts (Drescher, Ruf, Calsa, Carrer, & Bock, 2000). Ycf1 products are essential for cell survival (Kikuchi et al., 2013), and it is among the four genes (together with *matK*,* ndhF*, and *ccsA*) in which single‐nucleotide variations and insertion/deletion frequencies were clearly higher than average, showing a signature of positive selection (Daniell, Lin, Yu, & Chang, 2016). Ycf1 was also identified as the most promising plastid DNA barcode for land plants (Dong et al., 2015). N8 is located in the coding region of rpoB, which encodes the beta subunit of a multisubunit RNA polymerase (Little & Hallick, 1988). RpoB was also identified as a marker suitable for phylogenetic study due to its relatively high substitution rate in this sequence (Olmstead & Palmer, 1994). Our study thus provides more evidence of SSR‐mediated insertion/deletion variation in two highly variable chloroplast genes (ycf1 and rpoB) in Cupressaceae species.

### 4.3 CpSSR and its application in *P. orientalis*

Pollen dispersal is an important mechanism for long‐distance gene flow in conifers (Adams, 1992) and potentially plays a major role in maintaining genetic diversity (El‐Kassaby & Davidson, 1991; O'Connell, Mosseler, & Rajora, 2007) and, in turn, promoting adaptive evolution and positive response to rapid climate change (Kremer et al., 2012). Paternity analysis and tracking the origin of pollen also have significant implication for studies of mating systems, conservation genetics, and breeding (Chaisurisri & El‐Kassaby, 1994; El‐Kassaby, 1995). SSRs have already been recognized as an efficient means for paternity analysis in conifer species (Cato & Richardson, 1996; El‐Kassaby et al., 2011). To enrich the genetic toolkit, we developed a set of cpSSRs for the Cupressaceae and evaluated their application in population genetic studies by analyzing a core breeding population of *P. orientalis*. In the present study, 86 trees shared 28 genotypes over 10 polymorphic cpSSR loci, but each individual owns unique fingerprint by adding the polymorphic information of nuclear SSR. Low levels of differentiation among the 192 sampled trees had already been revealed in a previous study in which 10 polymorphic nuclear SSRs were used (Jin et al., 2016); we also obtained similar results with cpSSR, and the results of these two primers were also consistent with the above results. It has been suggested that cpSSR markers represent ideal molecular tools to complement nuclear genetic markers (Huang et al., 2015) in investigations of population genetics and biogeography and to unravel the genetic relationships of closely related species. Further analysis can be carried on combining the cpSSR with nuclear SSR*, as* these primers have significant discrimination rate on individual identification. Finally, the utility of the developed cpSSRs along with the available nuclear SSRs in paternity assignment and pedigree reconstruction cannot be understated, specifically when applied in marker‐assisted breeding schemes that aimed at simplifying the breeding process and speeding generation turnover for capturing greater gains (El‐Kassaby & Lstibůrek, 2009; El‐Kassaby et al., 2011).

## 5 CONCLUSION

We developed 26 polymorphic cpSSR markers for Cupressaceae species. The evaluation with 192 *P. orientalis* samples of a core breeding population demonstrated moderate confidence of individual identification, but significantly high discrimination rate was attained after combining with 10 nuclear SSRs. The high degree of interspecific transferability and polymorphism of the developed cpSSRs proved that cpSSRs are a powerful tool for population genetics and breeding of conifer species.

## CONFLICT OF INTEREST

None declared.

## AUTHOR CONTRIBUTIONS

Conceived and designed the experiments: JFM. Performed the experiments: LSH YJ. Analyzed the data: YQS YJ XGH. Contributed reagents/materials/analysis tools: QG FLG XLY JJZ. Wrote the paper: LSH JFM YAEK.

## DATA ACCESSIBILITY

Primers designed and evaluated: main body of text (Table 2).

## References

[ece34053-bib-0001] Adams, W. T. (1992). Gene dispersal within forest tree populations. New Forests, 6, 217–240. https://doi.org/10.1007/BF00120646

[ece34053-bib-0002] Birky, C. W. (1995). Uniparental inheritance of mitochondrial and chloroplast genes: Mechanisms and evolution. Proceedings of the National Academy of Sciences, 92, 11331–11338. https://doi.org/10.1073/pnas.92.25.11331 10.1073/pnas.92.25.11331 PMC40394 8524780

[ece34053-bib-0003] Cato, S. , & Richardson, T. (1996). Inter‐and intraspecific polymorphism at chloroplast SSR loci and the inheritance of plastids in *Pinus radiata* D. Don. Theoretical and Applied Genetics, 93, 587–592. https://doi.org/10.1007/BF00417952 24162352 10.1007/BF00417952

[ece34053-bib-0004] Chaisurisri, K. , & El‐Kassaby, Y. A. (1994). Genetic diversity in a seed production population vs. natural populations of Sitka Spruce. Biodiversity & Conservation, 3, 512–523. https://doi.org/10.1007/BF00115157

[ece34053-bib-0005] Cheng, Y. , De Vicente, M. C. , Meng, H. , Guo, W. , Tao, N. , & Deng, X. (2005). A set of primers for analyzing chloroplast DNA diversity in *Citrus* and related genera. Tree Physiology, 25, 661–672. https://doi.org/10.1093/treephys/25.6.661 15805086 10.1093/treephys/25.6.661

[ece34053-bib-0006] Cheng, W. C. , & Fu, L. K. (1978). Flora Reipublicae Popularis Sinicae.Gymnospermae, 7, 321–322.

[ece34053-bib-0007] Chu, J. M. (2012). Absorption and concentration effects of evergreen species (*Pinus tabuliformis* and *Platycladus orientalis*) to typical pollutants. Journal of Meteorology and Environment, 28, 15–20.

[ece34053-bib-0008] Daniell, H. , Lin, C. S. , Yu, M. , & Chang, W. J. (2016). Chloroplast genomes: Diversity, evolution, and applications in genetic engineering. Genome Biology, 17, 134 https://doi.org/10.1186/s13059-016-1004-2 27339192 10.1186/s13059-016-1004-2 PMC4918201

[ece34053-bib-0009] Dong, W. , Xu, C. , Li, C. , Sun, J. , Zuo, Y. , Shi, S. , … Zhou, S. (2015). Ycf1, the most promising plastid DNA barcode of land plants. Scientific Reports, 5(5), 8348 https://doi.org/10.1038/srep08348 25672218 10.1038/srep08348 PMC4325322

[ece34053-bib-0010] Drescher, A. , Ruf, S. , Calsa, T. , Carrer, H. , & Bock, R. (2000). The two largest chloroplast genome‐encoded open reading frames of higher plants are essential genes. Plant Journal, 22, 97–104. https://doi.org/10.1046/j.1365-313x.2000.00722.x 10792825 10.1046/j.1365-313x.2000.00722.x

[ece34053-bib-0011] Ebert, D. , & Peakall, R. O. D. (2009). Chloroplast simple sequence repeats (cpSSRs): Technical resources and recommendations for expanding cpSSR discovery and applications to a wide array of plant species. Molecular Ecology Resources, 9, 673–690. https://doi.org/10.1111/j.1755-0998.2008.02319.x 21564725 10.1111/j.1755-0998.2008.02319.x

[ece34053-bib-0012] El‐Kassaby, Y. A. (1995). Evaluation of the tree‐improvement delivery system: Factors affecting genetic potential. Tree Physiology, 15, 545–550. https://doi.org/10.1093/treephys/15.7-8.545 14965941 10.1093/treephys/15.7-8.545

[ece34053-bib-0013] El‐Kassaby, Y. A. , Cappa, E. P. , Liewlaksaneeyanawin, C. , Klápště, J. , & Lstibůrek, M. (2011). Breeding without breeding: Is a complete pedigree necessary for efficient breeding? PLoS ONE, 6, e25737 https://doi.org/10.1371/journal.pone.0025737 21991342 10.1371/journal.pone.0025737 PMC3185014

[ece34053-bib-0014] El‐Kassaby, Y. A. , & Davidson, R. (1991). Impact of pollination environment manipulation on the apparent outcrossing rate in a Douglas‐fir seed orchard. Heredity, 66, 55–59. https://doi.org/10.1038/hdy.1991.7

[ece34053-bib-0015] El‐Kassaby, Y. A. , & Lstibůrek, M. (2009). Breeding without breeding. Genetics Research, 91, 111–120. https://doi.org/10.1017/S001667230900007X 19393127 10.1017/S001667230900007X

[ece34053-bib-0016] Frazer, K. A. , Pachter, L. , Poliakov, A. , & Rubin, E. M. , Dubchak, I. (2012). VISTA: Computational tools for comparative genomics. Nucleic Acids Research, 32, W273–W279. 10.1093/nar/gkh458 PMC441596 15215394

[ece34053-bib-0017] Guo, W. , Grewe, F. , Cobo‐Clark, A. , Fan, W. , Duan, Z. , Adams, R. P. , … Mower, J. P. (2014). Predominant and substoichiometric isomers of the plastid genome coexist within Juniperus plants and have shifted multiple times during cupressophyte evolution. Genome Biology & Evolution, 6, 580–590. https://doi.org/10.1093/gbe/evu046 24586030 10.1093/gbe/evu046 PMC3971597

[ece34053-bib-0018] Hu, X. G. , Jin, Y. Q. , Wang, X. R. , Mao, J. F. , & Li, Y. (2015). Predicting impacts of future climate change on the distribution of the widespread conifer *Platycladus orientalis* . PLoS ONE, 10, e0132326 https://doi.org/10.1371/journal.pone.0132326 26132163 10.1371/journal.pone.0132326 PMC4488561

[ece34053-bib-0019] Huang, J. , Yang, X. , Zhang, C. , Yin, X. , Liu, S. , & Li, X. (2015). Development of chloroplast microsatellite markers and analysis of chloroplast diversity in Chinese Jujube (*Ziziphus jujuba* Mill.) and Wild Jujube (*Ziziphus acidojujuba* Mill.). PLoS ONE, 10, e0134519 https://doi.org/10.1371/journal.pone.0134519 26406601 10.1371/journal.pone.0134519 PMC4583483

[ece34053-bib-0020] Huson, D. H. (1998). SplitsTree: Analyzing and visualizing evolutionary data. Bioinformatics, 14, 68–73. https://doi.org/10.1093/bioinformatics/14.1.68 9520503 10.1093/bioinformatics/14.1.68

[ece34053-bib-0021] Ihaka, R. , & Gentleman, R. (1996). R: A language for data analysis and graphics. Journal of Computational and Graphical Statistics, 5, 299–314.

[ece34053-bib-0022] Jiang, P. , Shi, J. , Niu, P. X. , & Yue, L. U. (2009). Effect on activities of defensive enzymes and MDA content in leaves of *Platycladus orientalis* under naturally decreasing temperature. Journal of Shihezi University, 127, 487–493.

[ece34053-bib-0023] Jin, Y. , Ma, Y. , Wang, S. , Hu, X. G. , Huang, L. S. , Li, Y. , … Mao, J. F. (2016). Genetic evaluation of the breeding population of a valuable reforestation conifer *Platycladus orientalis* (Cupressaceae). Scientific Reports, 6(6), 34821 https://doi.org/10.1038/srep34821 27721449 10.1038/srep34821 PMC5056456

[ece34053-bib-0024] Jombart, T. (2008). Adegenet: A R package for the multivariate analysis of genetic markers. Bioinformatics, 24, 1403–1405. https://doi.org/10.1093/bioinformatics/btn129 18397895 10.1093/bioinformatics/btn129

[ece34053-bib-0025] Kikuchi, S. , Bédard, J. , Hirano, M. , Hirabayashi, Y. , Oishi, M. , Imai, M. , … Nakai, M. (2013). Uncovering the protein translocon at the chloroplast inner envelope membrane. Science, 339, 571–574. https://doi.org/10.1126/science.1229262 23372012 10.1126/science.1229262

[ece34053-bib-0026] Kremer, A. , Ronce, O. , Robledo‐Arnuncio, J. J. , Guillaume, F. , Bohrer, G. , Nathan, R. , … Kuparinen, A. (2012). Long‐distance gene flow and adaptation of forest trees to rapid climate change. Ecology Letters, 15, 378–392. https://doi.org/10.1111/j.1461-0248.2012.01746.x 22372546 10.1111/j.1461-0248.2012.01746.x PMC3490371

[ece34053-bib-0027] Li, H. , Guo, Q. , & Zheng, W. (2016). The complete chloroplast genome of *Cupressus gigantea*, an endemic conifer species to Qinghai‐Tibetan Plateau. Mitochondrial DNA, 27, 3743–3744. https://doi.org/10.3109/19401736.2015.1079885 26359779 10.3109/19401736.2015.1079885

[ece34053-bib-0028] Li, X. P. , He, Y. P. , & Ren, Q. F. (2011). Water stress experiments of *Platycladus orientalis* and *Pinus tablaeformis* young trees. Forest Research, 24, 91–96.

[ece34053-bib-0029] Little, M. C. , & Hallick, R. B. (1988). Chloroplast rpoA, rpoB, and rpoC genes specify at least three components of a chloroplast DNA‐dependent RNA polymerase active in tRNA and mRNA transcription. The Journal of Biological Chemistry, 263, 14302–14307. 3049574

[ece34053-bib-0030] Mayor, C. , Brudno, M. , Schwartz, J. R. , Poliakov, A. , Rubin, E. M. , Frazer, K. A. , … Dubchak, I. (2000). VISTA: Visualizing global DNA sequence alignments of arbitrary length. Bioinformatics, 16, 1046–1047. https://doi.org/10.1093/bioinformatics/16.11.1046 11159318 10.1093/bioinformatics/16.11.1046

[ece34053-bib-0031] O'Connell, L. M. , Mosseler, A. , & Rajora, O. P. (2007). Extensive long‐distance pollen dispersal in a fragmented landscape maintains genetic diversity in White Spruce. Journal of Heredity, 98, 640–645. https://doi.org/10.1093/jhered/esm089 17981919 10.1093/jhered/esm089

[ece34053-bib-0032] Olmstead, R. G. , & Palmer, J. D. (1994). Chloroplast DNA systematics: A review of methods and data analysis. American Journal of Botany, 81, 1205–1224. https://doi.org/10.1002/j.1537-2197.1994.tb15615.x

[ece34053-bib-0033] Pan, L. , Li, Y. , Guo, R. , Wu, H. , Hu, Z. , & Chen, C. (2014). Development of 12 chloroplast microsatellite markers in *Vigna unguiculata* (Fabaceae) and amplification in *Phaseolus vulgaris* . Applications in Plant Sciences, 2, 1300075. 10.3732/apps.1300075 PMC4103102 25202608

[ece34053-bib-0034] Peakall, R. , & Smouse, P. E. (2012). GenAlEx 6.5: Genetic analysis in Excel. Population genetic software for teaching and research—an update. Bioinformatics, 28, 2537–2539. https://doi.org/10.1093/bioinformatics/bts460 22820204 10.1093/bioinformatics/bts460 PMC3463245

[ece34053-bib-0035] Powell, W. , Machray, G. C. , & Provan, J. (1996). Polymorphism revealed by simple sequence repeats. Trends in Plant Science, 1, 215–222. https://doi.org/10.1016/S1360-1385(96)86898-0

[ece34053-bib-0036] Powell, W. , Morgante, M. , Andre, C. , McNicol, J. W. , Machray, G. C. , Doyle, J. J. , … Rafalski, J. A. (1995). Hypervariable microsatellites provide a general source of polymorphic DNA markers for the Chloroplast genome. Current Biology, 5, 1023–1029. https://doi.org/10.1016/S0960-9822(95)00206-5 8542278 10.1016/s0960-9822(95)00206-5

[ece34053-bib-0037] Powell, W. , Morgante, M. , McDevitt, R. , Vendramin, G. , & Rafalski, J. (1995). Polymorphic simple sequence repeat regions in chloroplast genomes: Applications to the population genetics of pines. Proceedings of the National Academy of Sciences of the United States of America, 92, 7759–7763. https://doi.org/10.1073/pnas.92.17.7759 7644491 10.1073/pnas.92.17.7759 PMC41225

[ece34053-bib-0038] Provan, J. , Powell, W. , & Hollingsworth, P. (2001). Chloroplast microsatellites: New tools for studies in plant ecology and evolution. Trends in Ecology & Evolution, 16, 142–147. https://doi.org/10.1016/S0169-5347(00)02097-8 11179578 10.1016/s0169-5347(00)02097-8

[ece34053-bib-0039] Rambaut, A. (2012). FigTree v1. 4.0. A graphical viewer of phylogenetic trees. Retrieved from http://tree.bio.ed.ac.uk/software/figtree/

[ece34053-bib-0040] Rozen, S. , & Skaletsky, H. (1999). Primer3 on the WWW for general users and for biologist programmers. Bioinformatics Methods and Protocols, 132, 365–386. https://doi.org/10.1385/1592591922 10.1385/1-59259-192-2:365 10547847

[ece34053-bib-0041] Sakaguchi, S. , Tsumura, Y. , Crisp, M. D. , Bowman, D. M. J. S. , & Isagi, Y. (2014). Genetic evidence for paternal inheritance of the chloroplast in four Australian *Callitris* species (Cupressaceae). Journal of Forest Research, 19, 244–248. https://doi.org/10.1007/s10310-012-0384-8

[ece34053-bib-0042] Schlotterer, C. , & Tautz, D. (1992). Slippage synthesis of simple sequence DNA. Nucleic Acids Research, 20, 211–215. https://doi.org/10.1093/nar/20.2.211 1741246 10.1093/nar/20.2.211 PMC310356

[ece34053-bib-0043] Schuler, G. D. (1997). Sequence mapping by electronic PCR. Genome Research, 7, 541–550. https://doi.org/10.1101/gr.7.5.541 9149949 10.1101/gr.7.5.541 PMC310656

[ece34053-bib-0044] Vendramin, G. G. , Lelli, L. , Rossi, P. , & Morgante, M. (1996). A set of primers for the amplification of 20 chloroplast microsatellites in Pinaceae. Molecular Ecology, 5, 595–598. https://doi.org/10.1111/j.1365-294X.1996.tb00353.x 8794566 10.1111/j.1365-294x.1996.tb00353.x

[ece34053-bib-0045] Wang, X. W. , & Wang, L. (2016). GMATA: An integrated software package for genome‐scale SSR mining, marker development and viewing. Frontiers in Plant Science, 7, 1350. 27679641 10.3389/fpls.2016.01350 PMC5020087

[ece34053-bib-0046] Wang, Y. S. , Xing, S. Y. , Tang, H. X. , & Feng, D. Q. (2011). Genetic diversity of *Platycladus orientalis* provenances. Scientia Silvae Sinicae, 47, 91–96.

[ece34053-bib-0047] Wilberg, M. J. , & Dreher, B. P. (2004). Genecap: A program for analysis of multilocus genotype data for non‐invasive sampling and capture‐recapture population estimation. Molecular Ecology Notes, 4, 783–785. https://doi.org/10.1111/j.1471-8286.2004.00797.x

[ece34053-bib-0048] Xie, C. Y. , Dancik, B. P. , & Yeh, F. C. (1992). Genetic structure of *Thuja orientalis* . Biochemical Systematics and Ecology, 20, 433–441. https://doi.org/10.1016/0305-1978(92)90083-P

