# Supplementary figures and images for: Development of high transferability cpSSR markers for individual identification and genetic investigation in Cupressaceae species
Source: Ecol Evol. 2018 Apr 20;8(10):4967–77. doi: 10.1002/ece3.4053 (PMC5980425; doi:10.1002/ece3.4053)

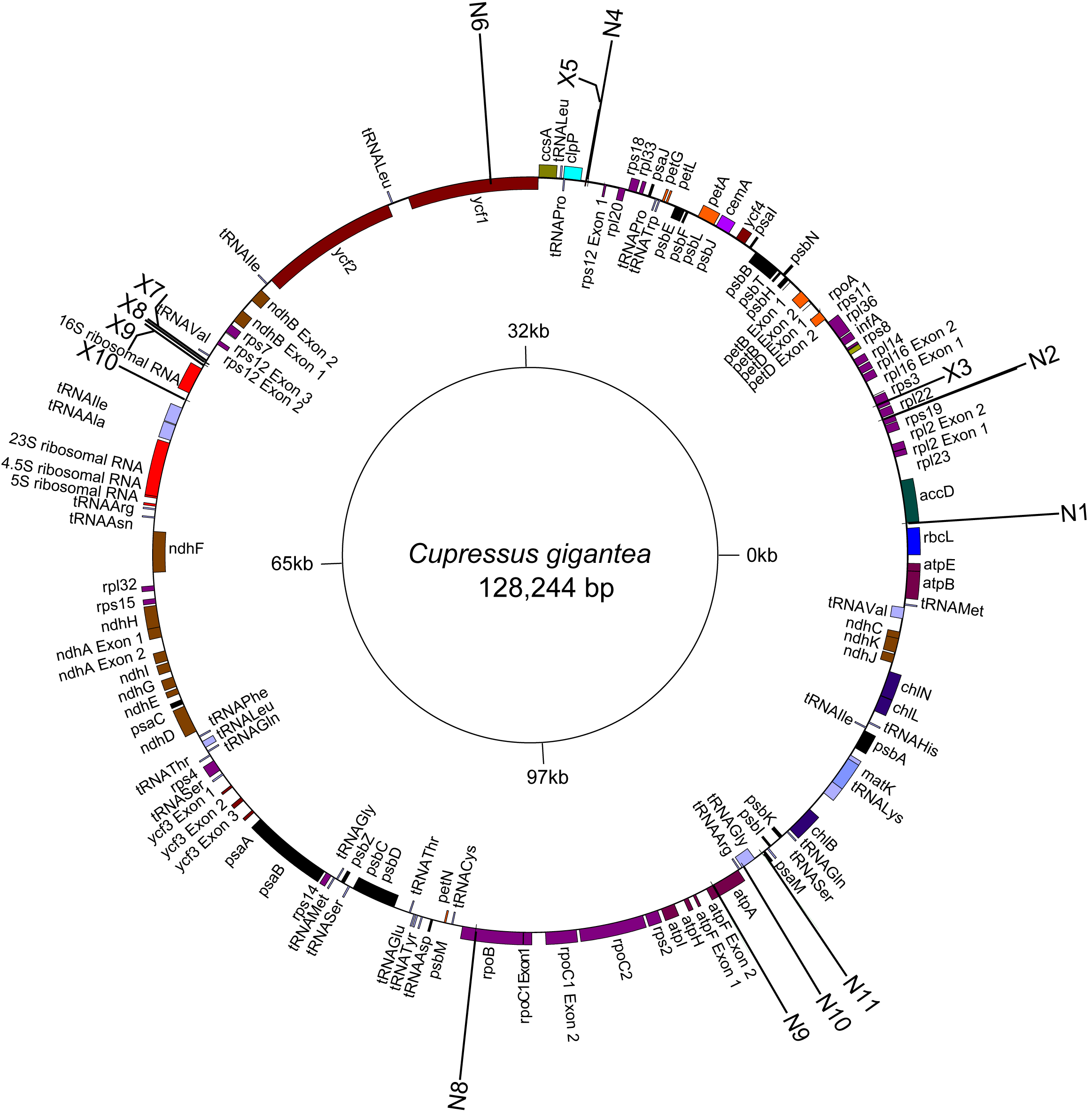

Supplement: Supplementary file 1 [file ECE3-8-4967-s001.tif]

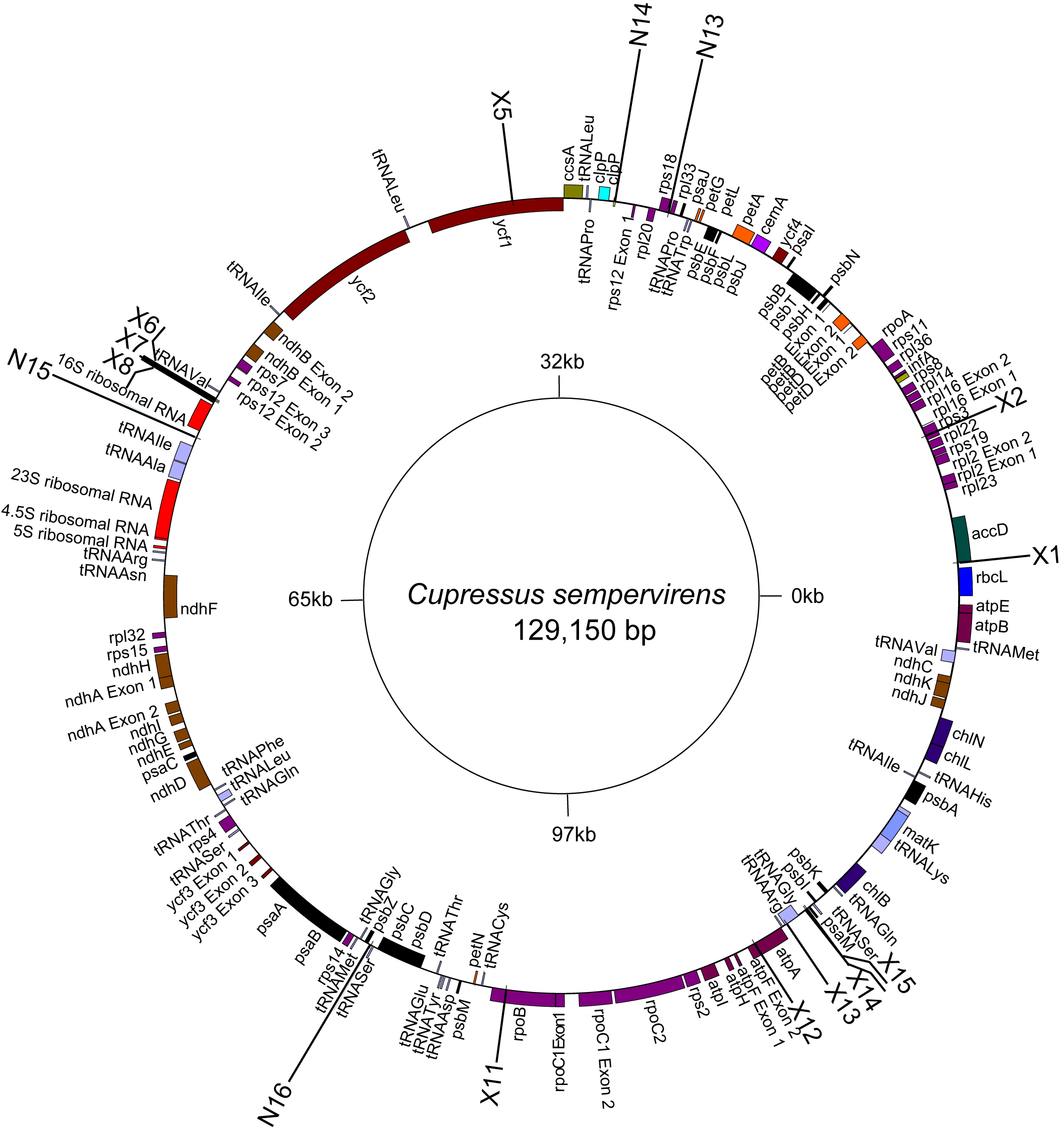

Supplement: Supplementary file 2 [file ECE3-8-4967-s002.tif]

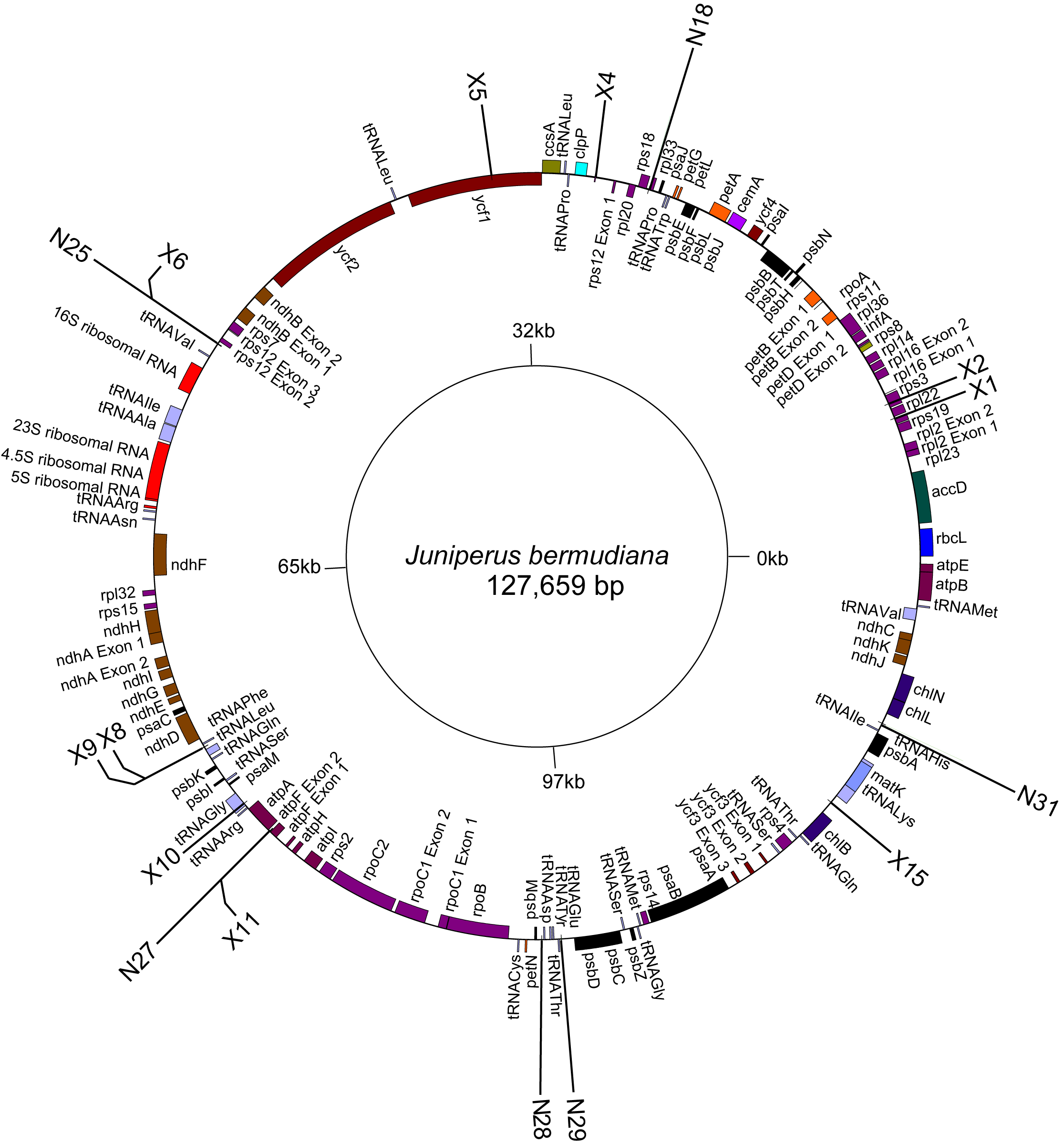

Supplement: Supplementary file 3 [file ECE3-8-4967-s003.tif]

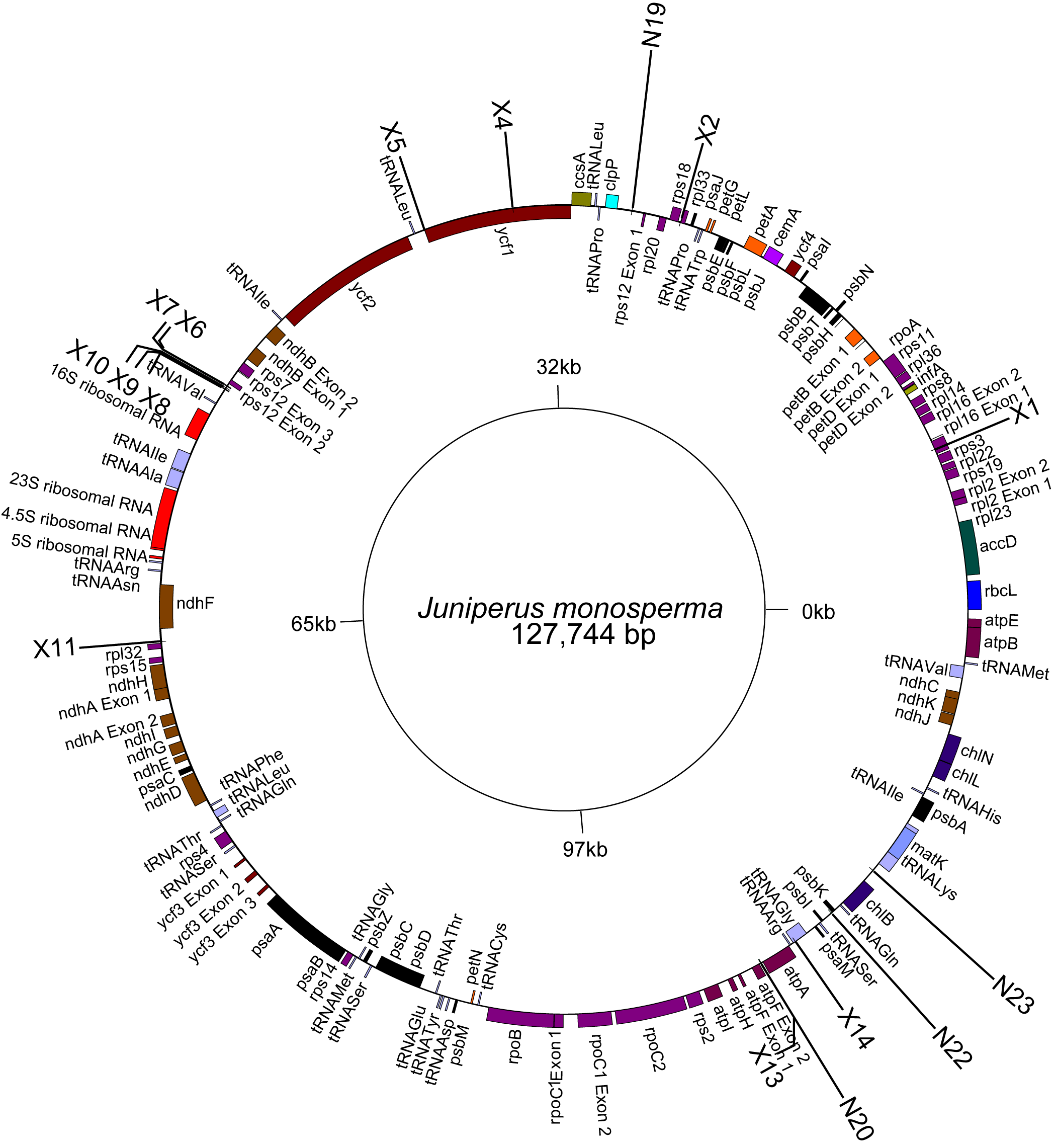

Supplement: Supplementary file 4 [file ECE3-8-4967-s004.tif]

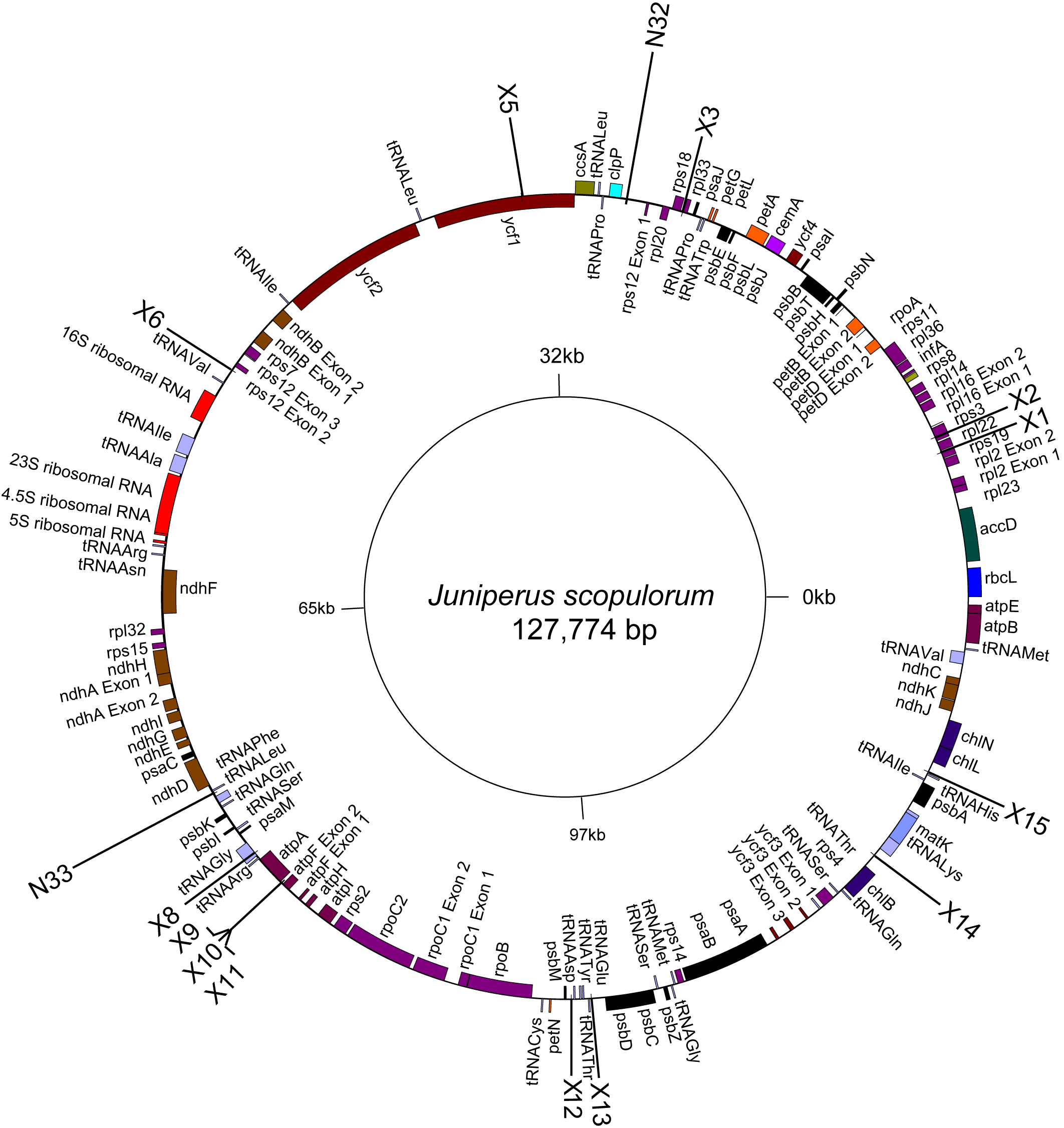

Supplement: Supplementary file 5 [file ECE3-8-4967-s005.tif]

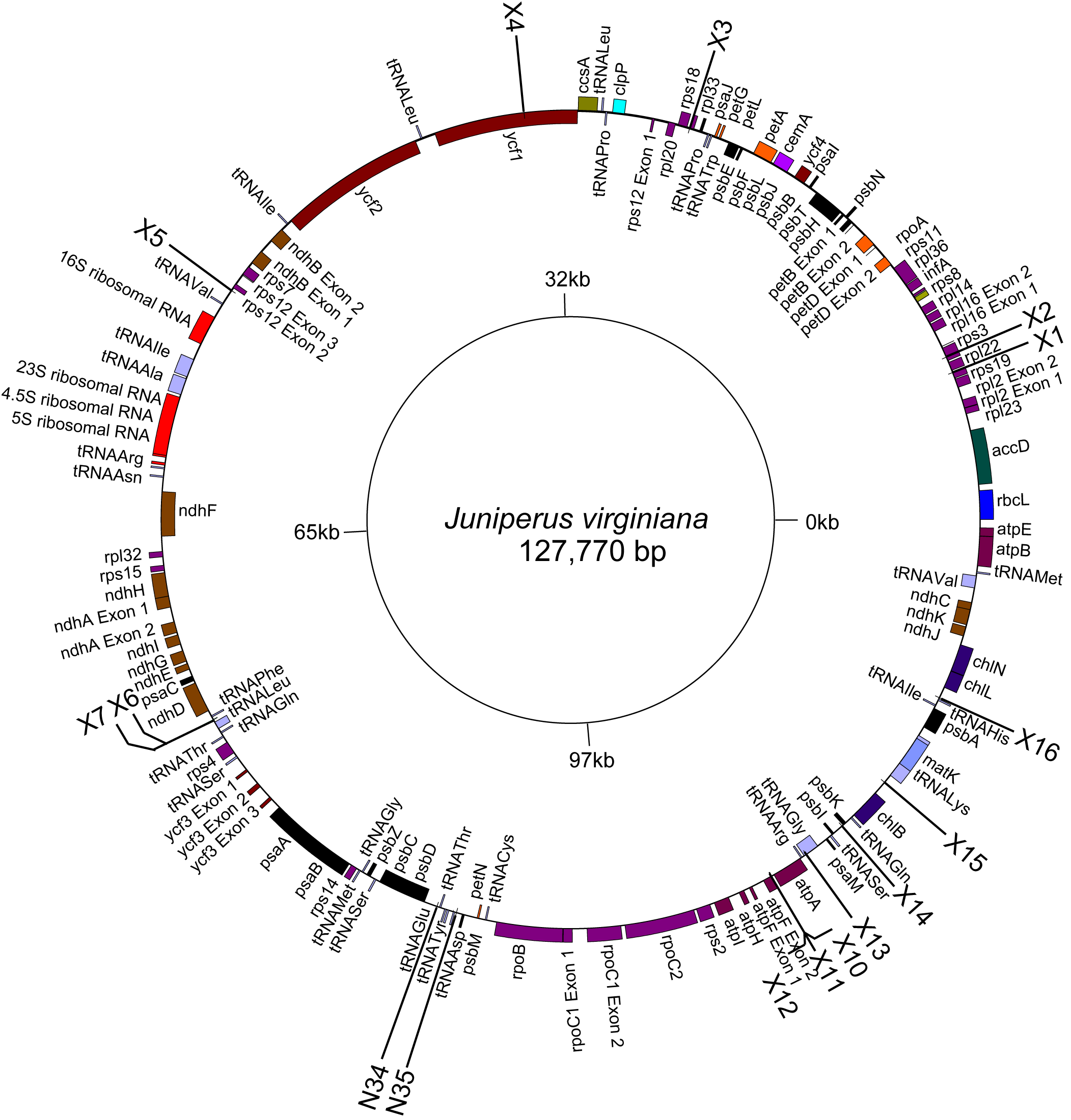

Supplement: Supplementary file 6 [file ECE3-8-4967-s006.tif]

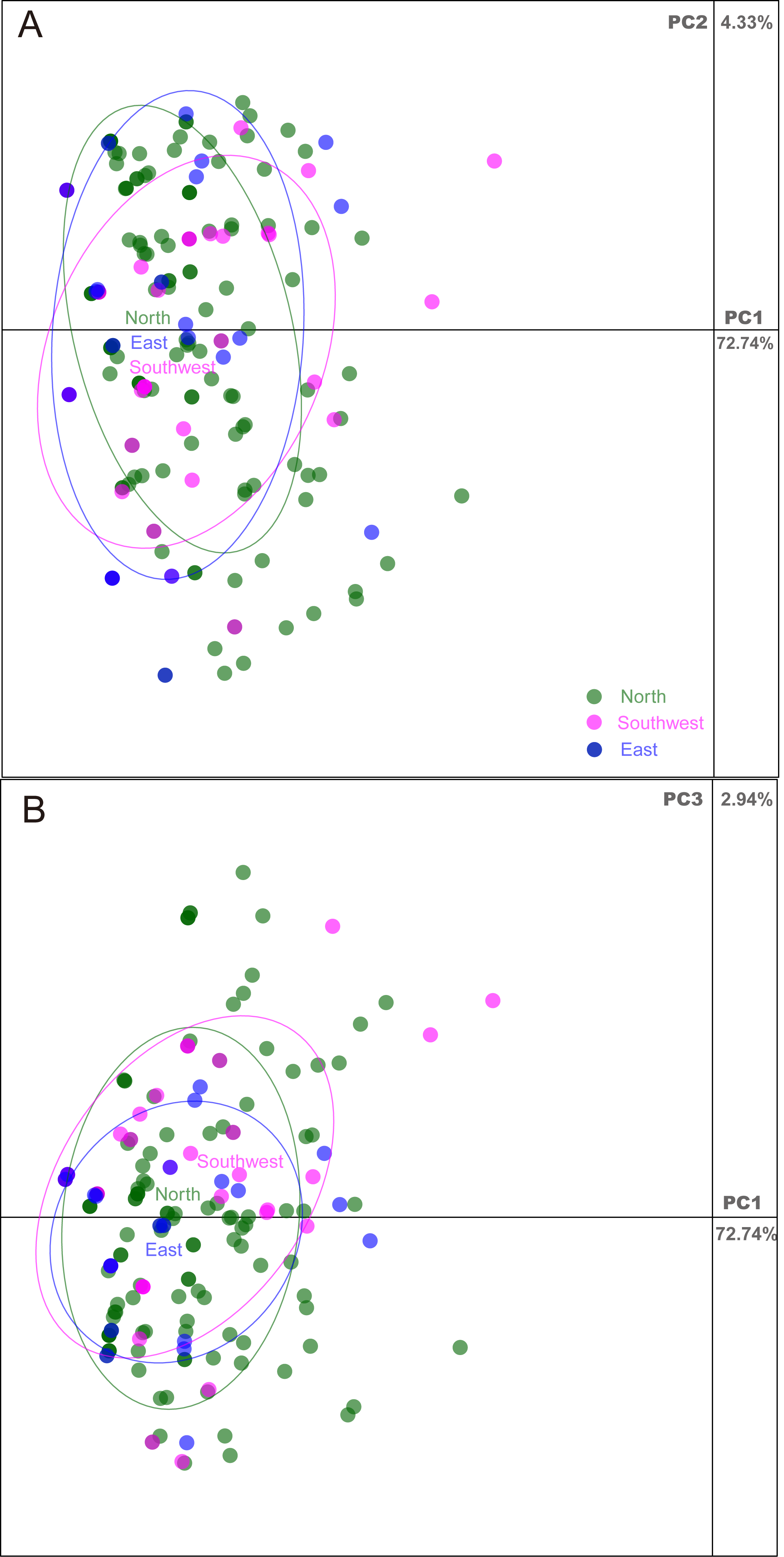

Supplement: Supplementary file 7 [file ECE3-8-4967-s007.tif]

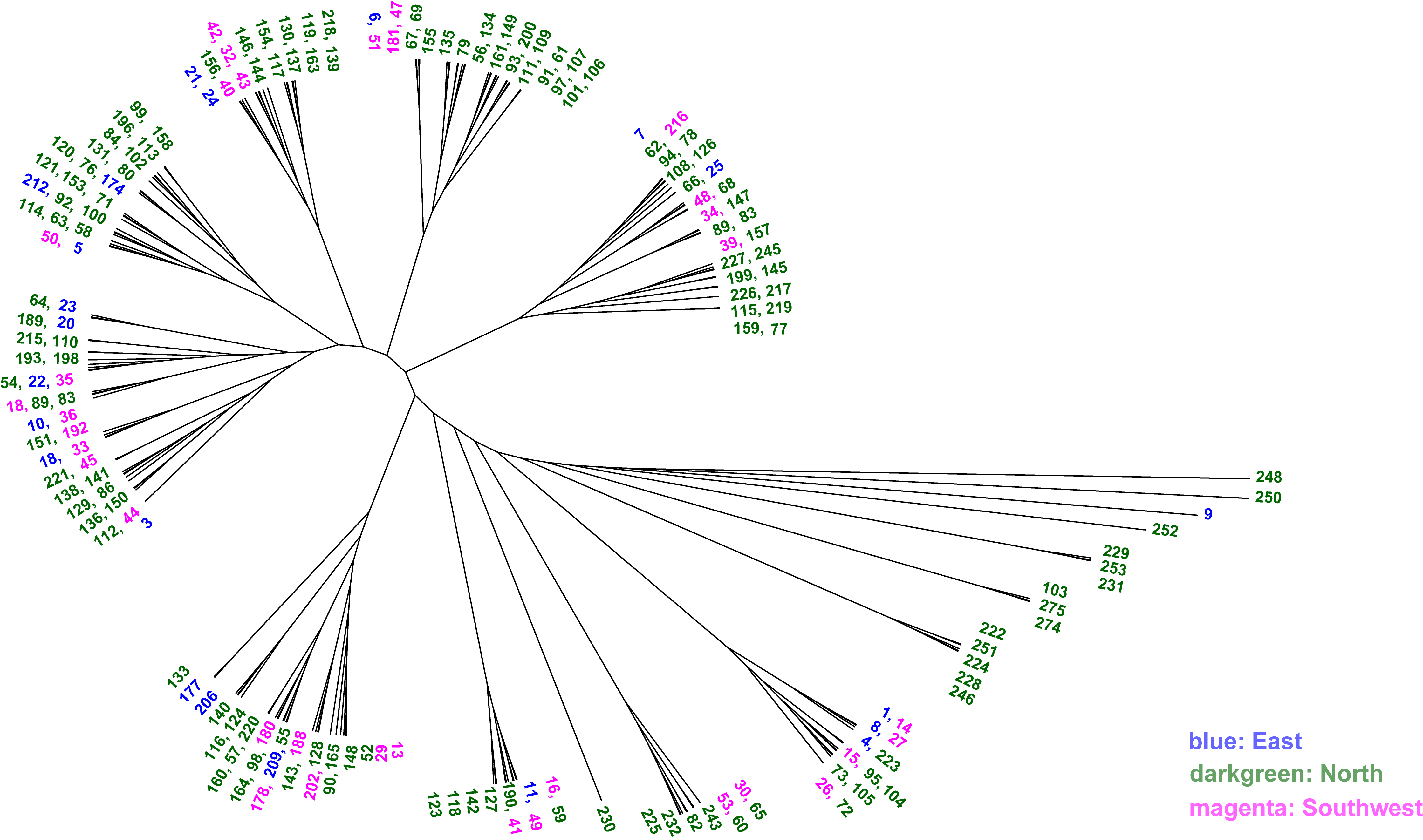

Supplement: Supplementary file 8 [file ECE3-8-4967-s008.tif]
