# Supplementary material for: Development of high transferability cpSSR markers for individual identification and genetic investigation in Cupressaceae species
Source: Ecol Evol. 2018 Apr 20;8(10):4967–77. doi: 10.1002/ece3.4053 (PMC5980425; doi:10.1002/ece3.4053)
Supplement: Supplementary file 9 [file ECE3-8-4967-s009.docx]

Development of high transferability cpSSR markers for individual identification and genetic investigation in Cupressaceae species

LI-SHA HUANG^1‡^, YAN-QIANG SUN^1‡^, YUQING JIN^1‡^, QIONG GAO^1^, XIAN-GE HU^1^, FU-LING GAO^2^, XIAO-LEI YANG^3^, JI-JUN ZHU^3^, YOUSRY A. EL-KASSABY^4^, JIAN-FENG MAO^1*^

*^1^* *Beijing Advanced Innovation Center for Tree Breeding By Molecular Design, National Engineering Laboratory for Tree Breeding, Key Laboratory of Genetics and Breeding in Forest Trees and Ornamental Plants, Ministry of Education, College of Biological Sciences and Technology, Beijing Forestry University, Beijing 100083, China.*

*^2^ Economic Forest and Seeding Management Station in Henan Province, Zhengzhou 450000, China.*

*^3^ National Tree Breeding Station for Platycladus orientalis in Jiaxian, Forest Farm of Jiaxian County, Henan 467100, China.*

*^4^ Department of Forest and Conservation Sciences, Faculty of Forestry, The University of British Columbia, Vancouver, British Columbia, Vancouver V6T 1Z4, Canada.*

^‡^These authors contributed equally to this paper.

^*^**Correspondence to:** Dr. Jian-Feng Mao; E-mail: [jianfeng.mao@bjfu.edu.cn](mailto:jianfeng.mao@bjfu.edu.cn)

**Supplementary information**

**T****able S1** Characterization of 92 SSR loci detected in the chloroplast genomes of six species (*Cupressus gigantean*, *Cupressus sempervirens*, *Juniperus monosperma*, *Juniperus bermudiana*, *Juniperus scopulorum* and *Juniperus virgiana*).

**Table S2** E-PCR amplification of 26 pairs of primers against the chloroplast genomes of seven Cupressaceae species (*Cupressus jiangeensis*, *Calocedrus formosana*, *Thujopsis dolabrata*, *Thuja standishii*, *Cunninghamia lanceolata*, *Calocedrus macrolepis* and *Hesperocyparis glabra*).

**Fig. S1** Overview of SSR distribution in *Cupressus gigantean* chloroplast genome. The positions of the short line marked by the strings initiated with “X” and “N” are indicating the positions with SSR loci detected. SSR loci that were experimentally evaluated have tags initiated with “N”.

**Fig. S2** Overview of SSR distribution in *Cupressus sempervirens* chloroplast genome. The positions of the short line marked by the strings initiated with “X” and “N” are indicating the positions with SSR loci detected. SSR loci that were experimentally evaluated have tags initiated with “N”.

**Fig. S3** Overview of SSR distribution in *Juniperus bermudiana* chloroplast genome. The positions of the short line marked by the strings initiated with “X” and “N” are indicating the positions with SSR loci detected. SSR loci that were experimentally evaluated have tags initiated with “N”.

**Fig. S4** Overview of SSR distribution in *Juniperus monosperma* chloroplast genome. The positions of the short line marked by the strings initiated with “X” and “N” are indicating the positions with SSR loci detected. SSR loci that were experimentally evaluated have tags initiated with “N”.

**Fig. S5** Overview of SSR distribution in *Juniperus scopulorum* chloroplast genome. The positions of the short line marked by the strings initiated with “X” and “N” are indicating the positions with SSR loci detected. SSR loci that were experimentally evaluated have tags initiated with “N”.

**Fi****g. S6** Overview of SSR distribution in *Juniperus virginiana* chloroplast genome. The positions of the short line marked by the strings initiated with “X” and “N” are indicating the positions with SSR loci detected. SSR loci that were experimentally evaluated have tags initiated with “N”.

**Fig. S7** Principal Component Analysis (PCA) of *P. orientalis* populations’ plastomes based on the polymorphism information of 10 cpSSRs.

**Fig. S8** Dendrogram of *P. orientalis* plastomes. The trees was marked with different colors to show their geographic information, showing no clear clusters related to their geological origins.

**Table S1** Characterization of 92 SSR loci detected in the chloroplast genomes of six species (*Cupressus gigantean*, *Cupressus sempervirens*, *Juniperus monosperma*, *Juniperus bermudiana*, *Juniperus scopulorum* and *Juniperus virgiana*).

| **Species** | **Id** | **Sequence Length (bp)** | **Start Position** | **End Position** | **Repetitions** | | **Motif** |
| --- | --- | --- | --- | --- | --- | --- | --- |
| *Juniperus bermudiana* | X1 | 127659 | 7486 | 7497 | | 6 | AC |
| *Juniperus bermudiana* | X2 | 127659 | 8318 | 8327 | | 5 | AT |
| *Juniperus bermudiana* | N18 | 127659 | 25905 | 25914 | | 5 | AT |
| *Juniperus bermudiana* | X4 | 127659 | 28788 | 28832 | | 5 | AAGGACAAA |
| *Juniperus bermudiana* | X5 | 127659 | 34323 | 34340 | | 6 | TTC |
| *Juniperus bermudiana* | X6 | 127659 | 51875 | 51884 | | 5 | TA |
| *Juniperus bermudiana* | N25 | 127659 | 51909 | 51918 | | 5 | TA |
| *Juniperus bermudiana* | X8 | 127659 | 74398 | 74407 | | 5 | AT |
| *Juniperus bermudiana* | X9 | 127659 | 74409 | 74418 | | 5 | AT |
| *Juniperus bermudiana* | X10 | 127659 | 78066 | 78075 | | 5 | TC |
| *Juniperus bermudiana* | X11 | 127659 | 80061 | 80072 | | 6 | AT |
| *Juniperus bermudiana* | N27 | 127659 | 80086 | 80109 | | 12 | TA |
| *Juniperus bermudiana* | N28 | 127659 | 95999 | 96008 | | 5 | CA |
| *Juniperus bermudiana* | N29 | 127659 | 97039 | 97050 | | 6 | AT |
| *Juniperus bermudiana* | X15 | 127659 | 113512 | 113529 | | 6 | AGA |
| *Juniperus bermudiana* | N31 | 127659 | 118510 | 118519 | | 5 | TA |
| *Juniperus monosperma* | X1 | 127744 | 8275 | 8284 | | 5 | AT |
| *Juniperus monosperma* | X2 | 127744 | 25988 | 25999 | | 6 | AT |
| *Juniperus monosperma* | N19 | 127744 | 28502 | 28516 | | 5 | TAA |
| *Juniperus monosperma* | X4 | 127744 | 34524 | 34544 | | 7 | TTC |
| *Juniperus monosperma* | X5 | 127744 | 39106 | 39119 | | 7 | TA |
| *Juniperus monosperma* | X6 | 127744 | 51967 | 51978 | | 6 | AT |
| *Juniperus monosperma* | X7 | 127744 | 52001 | 52012 | | 6 | AT |
| *Juniperus monosperma* | X8 | 127744 | 52089 | 52098 | | 5 | AT |
| *Juniperus monosperma* | X9 | 127744 | 52154 | 52188 | | 5 | ATATAGA |
| *Juniperus monosperma* | X10 | 127744 | 52190 | 52199 | | 5 | TA |
| *Juniperus monosperma* | X11 | 127744 | 65480 | 65493 | | 7 | AT |
| *Juniperus monosperma* | N20 | 127744 | 106139 | 106156 | | 9 | TA |
| *Juniperus monosperma* | X13 | 127744 | 106168 | 106179 | | 6 | AT |
| *Juniperus monosperma* | X14 | 127744 | 108204 | 108213 | | 5 | GA |
| *Juniperus monosperma* | N22 | 127744 | 110505 | 110516 | | 6 | AT |
| *Juniperus monosperma* | N23 | 127744 | 113509 | 113526 | | 6 | AGA |
| *Juniperus scopulorum* | X1 | 127774 | 7470 | 7481 | | 6 | AC |
| *Juniperus scopulorum* | X2 | 127774 | 8302 | 8311 | | 5 | AT |
| *Juniperus scopulorum* | X3 | 127774 | 25928 | 25937 | | 5 | AT |
| *Juniperus scopulorum* | N32 | 127774 | 28787 | 28831 | | 5 | AAGGACAAA |
| *Juniperus scopulorum* | X5 | 127774 | 34136 | 34150 | | 5 | TTC |
| *Juniperus scopulorum* | X6 | 127774 | 51700 | 51709 | | 5 | TA |
| *Juniperus scopulorum* | N33 | 127774 | 74291 | 74300 | | 5 | AT |
| *Juniperus scopulorum* | X8 | 127774 | 77945 | 77954 | | 5 | TC |
| *Juniperus scopulorum* | X9 | 127774 | 79964 | 79973 | | 5 | AT |
| *Juniperus scopulorum* | X10 | 127774 | 79975 | 79984 | | 5 | AT |
| *Juniperus scopulorum* | X11 | 127774 | 79998 | 80009 | | 6 | TA |

**Table S1** continued

| **Species** | **Id** | **Sequence Length (bp)** | **Start Position** | **End Position** | **Repetitions** | **Motif** |
| --- | --- | --- | --- | --- | --- | --- |

| *Juniperus scopulorum* | X12 | 127774 | 96150 | 96159 | 5 | CA |
| --- | --- | --- | --- | --- | --- | --- |
| *Juniperus scopulorum* | X13 | 127774 | 97163 | 97174 | 6 | AT |
| *Juniperus scopulorum* | X14 | 127774 | 113603 | 113620 | 6 | AGA |
| *Juniperus scopulorum* | X15 | 127774 | 118625 | 118634 | 5 | TA |
| *Juniperus virginiana* | X1 | 127770 | 7499 | 7510 | 6 | AC |
| *Juniperus virginiana* | X2 | 127770 | 8332 | 8341 | 5 | AT |
| *Juniperus virginiana* | X3 | 127770 | 25956 | 25965 | 5 | AT |
| *Juniperus virginiana* | X4 | 127770 | 34255 | 34269 | 5 | TTC |
| *Juniperus virginiana* | X5 | 127770 | 51772 | 51781 | 5 | TA |
| *Juniperus virginiana* | X6 | 127770 | 74260 | 74269 | 5 | AT |
| *Juniperus virginiana* | X7 | 127770 | 74270 | 74279 | 5 | TA |
| *Juniperus virginiana* | N34 | 127770 | 89064 | 89075 | 6 | TA |
| *Juniperus virginiana* | N35 | 127770 | 90080 | 90089 | 5 | TG |
| *Juniperus virginiana* | X10 | 127770 | 106227 | 106240 | 7 | AT |
| *Juniperus virginiana* | X11 | 127770 | 106255 | 106268 | 7 | AT |
| *Juniperus virginiana* | X12 | 127770 | 106269 | 106278 | 5 | TA |
| *Juniperus virginiana* | X13 | 127770 | 108289 | 108298 | 5 | GA |
| *Juniperus virginiana* | X14 | 127770 | 110565 | 110574 | 5 | AT |
| *Juniperus virginiana* | X15 | 127770 | 113592 | 113609 | 6 | AGA |
| *Juniperus virginiana* | X16 | 127770 | 118619 | 118628 | 5 | TA |
| *Cupressus sempervirens* | X1 | 129150 | 1718 | 1729 | 6 | AT |
| *Cupressus sempervirens* | X2 | 129150 | 8496 | 8505 | 5 | AT |
| *Cupressus sempervirens* | N13 | 129150 | 26674 | 26685 | 6 | TA |
| *Cupressus sempervirens* | N14 | 129150 | 29485 | 29565 | 9 | AAAAAGGAG |
| *Cupressus sempervirens* | X5 | 129150 | 34809 | 34823 | 5 | TCT |
| *Cupressus sempervirens* | X6 | 129150 | 53925 | 53936 | 6 | AT |
| *Cupressus sempervirens* | X7 | 129150 | 53993 | 54002 | 5 | TA |
| *Cupressus sempervirens* | X8 | 129150 | 54047 | 54056 | 5 | AT |
| *Cupressus sempervirens* | N15 | 129150 | 56168 | 56177 | 5 | TA |
| *Cupressus sempervirens* | N16 | 129150 | 86604 | 86617 | 7 | TA |
| *Cupressus sempervirens* | X11 | 129150 | 93988 | 94011 | 8 | AGA |
| *Cupressus sempervirens* | X12 | 129150 | 107286 | 107297 | 6 | TA |
| *Cupressus sempervirens* | X13 | 129150 | 109253 | 109262 | 5 | GA |
| *Cupressus sempervirens* | X14 | 129150 | 110482 | 110491 | 5 | AT |
| *Cupressus sempervirens* | X15 | 129150 | 110548 | 110559 | 6 | TA |
| *Cupressus gigantean* | N1 | 128244 | 1718 | 1727 | 5 | AT |
| *Cupressus gigantean* | N2 | 128244 | 7497 | 7508 | 6 | CA |
| *Cupressus gigantean* | X3 | 128244 | 8319 | 8328 | 5 | AT |
| *Cupressus gigantean* | N4 | 128244 | 28910 | 28933 | 6 | TAAG |
| *Cupressus gigantean* | X5 | 128244 | 29018 | 29062 | 5 | AAGAAGGAA |
| *Cupressus gigantean* | N6 | 128244 | 34248 | 34262 | 5 | TCT |
| *Cupressus gigantean* | X7 | 128244 | 53163 | 53174 | 6 | AT |
| *Cupressus gigantean* | X8 | 128244 | 53203 | 53214 | 6 | AT |
| *Cupressus gigantean* | X9 | 128244 | 53323 | 53346 | 12 | TA |
| *Cupressus gigantean* | X10 | 128244 | 55474 | 55485 | 6 | TA |
| *Cupressus gigantean* | N8 | 128244 | 93240 | 93263 | 8 | AGA |
| *Cupressus gigantean* | N9 | 128244 | 106493 | 106504 | 6 | TA |
| *Cupressus gigantean* | N10 | 128244 | 108455 | 108464 | 5 | GA |
| *Cupressus gigantean* | N11 | 128244 | 109674 | 109687 | 7 | AT |

**Table S2** E-PCR amplification of 26 pairs of primers against the chloroplast genomes of seven Cupressaceae species (*Cupressus jiangeensis*, *Calocedrus formosana*, *Thujopsis dolabrata*, *Thuja standishii*, *Cunninghamia lanceolata*, *Calocedrus macrolepis* and *Hesperocyparis glabra*).

| **Species** | **GenBank ID** | **Primer ID for successful amplification** |
| --- | --- | --- |
| *Cupressus jiangeensis* | MG596347.1 | N1,N2,N4,N6,N8,N9,N10,N11,N13,N16,N18,N19,N22,N25,N31,N33,N34,N35 |
| *Calocedrus formosana* | NC_023121.1 | N8,N9,N16,N20 |
| *Thujopsis dolabrata* | KX832628.1 | N2,N18,N29 |
| *Thuja standishii* | KX832627.1 | N1,N2,N9,N18,N34 |
| *Cunninghamia lanceolata* | NC_021437.1 | N9,N34 |
| *Calocedrus macrolepis* | KX832621.1 | N18 |
| *Hesperocyparis glabra* | KX832624.1 | N27,N29 |
